# Why is the data quality of hospital morbidity so heterogeneous? A nationwide survey of Portuguese clinical coders

**DOI:** 10.1186/s13690-026-01985-1

**Published:** 2026-07-07

**Authors:** João Vasco Santos, Iracy Silva Pimenta, Filipa Santos Martins, Vera Pinheiro, Tallys Kalynka Feldens, Fernando Lopes, Júlio Souza, Alberto Freitas

**Affiliations:** 1 MEDCIDS, Department of Community Medicine, Information and Health Decision Sciences, Faculty of Medicine, University of Porto, Porto, Portugal; 2 RISE-Health, Faculty of Medicine of the University of Porto, Rua Dr. Plácido da Costa, Porto, 4200-450 Portugal; 3 Public Health Unit, ULS Santo António, Porto, Portugal; 4 Department of Neurosciences and Mental Health, Faculty of Medicine, University of Porto, Porto, Portugal; 5 Public Health Unit, ULS Matosinhos, Matosinhos, Portugal; 6 Federal University of Paraná, Curitiba, Brazil; 7 Institute of Engineering – Polytechnic of Porto, Porto, Portugal

## Abstract

**Background:**

Hospital morbidity data are used for hospital reimbursement in many countries and are also critical for epidemiological profiling and health planning, both of which depend on data quality. Exploring the clinical coders' perspective is crucial for implementing targeted interventions in this field.

**Objective:**

To identify the main factors influencing the quality of health registries, the coding process and data quality, from the perspective of clinical coders in Portugal.

**Methods:**

We conducted a nationwide online survey of clinical coders, informed by prior focus groups and a literature review, to assess their perspectives on the quality of health records, the clinical coding process, and coded data.

**Results:**

A total of 162 responses were obtained. Respondents primarily used electronic health records, including discharge notes, clinical diaries, and surgical reports. Common problems in health records included "copy-paste" practices (67%) and the use of unspecified acronyms (52%). Incomplete records, inconsistent information, and the lack of support affected coding quality. Half agreed that a reduction in the number of coders contributed to delays. Financial incentives for quality, software availability, and frequent internal audits were identified as priority measures to improve data quality.

**Conclusion:**

This study highlights the importance of clinical documentation for coding accuracy. Despite the use of electronic health records and the transition to ICD-10-CM/PCS, difficulties such as “copy-paste” practices and the use of unspecified acronyms persist. Continuous training, standardisation of documentation practices, and collaboration between clinical coders and other health professionals are essential to ensure coding accuracy. These problems and potential improvements may affect national policymaking and initiatives such as the European Health Data Space and international health prioritisation.

**Supplementary Information:**

The online version contains supplementary material available at https://doi.org/10.1186/s13690-026-01985-1.

**Table:** 

Text box 1. Contributions to the literature
• Hospital morbidity data are widely used for reimbursement and health planning worldwide, yet few studies assess their quality from the perspective of clinical coders.
• This study provides a nationwide perspective on Portuguese clinical coders' perceptions of factors affecting data quality, including documentation practices, coder training, and process-related challenges.
• It highlights the persistence of problems such as "copy-paste" and unspecified acronyms in electronic health records, despite digitalisation.
• The findings underscore the need for standardised practices, training, and audits to improve coding quality across hospital settings.
• Results are timely for European initiatives, including the European Health Data Space.

## Introduction

Hospital morbidity data are an essential source of morbidity information, as they present demographic, clinical, and administrative data in a structured format. Nevertheless, often the primary purpose of these data is hospital reimbursement. Up until the end of 2023, Portuguese hospitals were mainly funded through Diagnosis-Related Groups (DRGs), which are clinically homogeneous categories with similar resource consumption, and were entirely dependent on the accuracy and completeness of hospital clinical coding [5]. In 2024, the national reform, with the generalisation of Local Health Units (ULS), introduced a shift in financing, merging hospitals and primary care into unified organisational structures funded through risk-adjusted capitation based on Adjusted Clinical Groups (ACGs). Despite this transition, the highest-quality and most complete morbidity information available in Portugal continues to derive from inpatient morbidity clinical coding, which remains essential for monitoring disease burden, planning services, and supporting policies and other secondary data uses.

A particular framing of the Portuguese system is that clinical coding is performed exclusively by trained physicians. Compared with previous studies, which were primarily based on small teams of health information managers, our study captures the largest Portugal-wide physician-coder sample to date and highlights system-specific barriers and facilitators that may differ from those in other settings.

As mentioned, the poor quality of coded data has implications for evaluating hospital production and activity, with repercussions for hospital financing. Moreover, data quality issues are an important constraint on geographical or temporal comparability, which may lead to imprecise estimates and biased planning despite efforts to identify and minimise sources of error.

Data quality can be compromised depending on several factors. In the Portuguese context, previous research on hospital morbidity data has highlighted that the quality of medical records and the interaction between physicians and coders are crucial to the accuracy of clinical coding [3]. Evidence from other settings indicates that health professionals’ knowledge and perceptions significantly influence the quality of medical records, thereby directly affecting clinical coding accuracy [43]. Lack of awareness and engagement with the coding system also contribute to poor data quality, underscoring the need for better training and involvement of all healthcare staff in the documentation process [40].

Nevertheless, clinical coders often face difficulties due to health professionals’ missing, incomplete, or nonspecific documentation of diagnostic information, which is frequently rife with errors, inaccuracies, and discrepancies. Different terminology, lack of communication between coders and physicians, variability in interpretation by different healthcare professionals, lack of identification of comorbidities and associated diagnoses are among the most frequently encountered causes of data quality problems in hospital databases [2, 6, 7, 10, 24, 44, 45]. The impact of transcription and abstraction errors on hospital DRG classification also highlights the need for accurate data entry [8].

To explore potential factors impacting coding quality and the aforementioned challenges, the use of focus groups as well as questionnaires to stakeholders involved in the process is frequent [1, 6, 10, 17, 24, 39, 44, 45]. However, in Europe, only a few papers using focus groups explored the coders’ perceptions [3, 4]. In the current study, these perceptions were further captured using a more comprehensive nationwide survey. Our goal was to explore and identify the main factors influencing the quality of health registries, coding process and data quality, from the perspective of clinical coders in Portugal.

### Clinical coding in Portugal

In Portugal, the clinical coding process is conducted by physician coders who receive specialised training for this specific role. This training is offered by the National School of Public Health, in partnership with the Central Administration of the Health System (*Administração Central do Sistema de Saúde*, ACSS). Coding activities can be performed part-time or full-time by physician coders, with the former being more common [4].

The clinical coding process in Portugal involves several stages, beginning with the review of patient health records – available in electronic or paper formats – to extract relevant information on diagnoses and procedures performed. Whether electronic or paper-based, health records constitute a key source of data on a patient’s health status, diseases, disease progression, procedures, treatment efficacy, and the quality of healthcare. The specific type of record used for coding may vary between hospitals, with some facilities coding from the discharge note, while others utilise the clinical diary or another type of record [3, 4].

After extracting the pertinent information from the health records, codes are assigned according to the International Classification of Diseases (ICD). Until the end of 2016, the Portuguese healthcare system used the Ninth Revision – Clinical Modification ICD-9-CM), at which point the Tenth Revision – Clinical Modification/Procedure Classification System (ICD-10-CM/PCS) was introduced. The transition to ICD-10-CM/PCS increased the complexity of the coding process due to the greater volume of codes, requiring additional coding time as coders adapted to the new system [3, 25].

Subsequently, assigned codes are entered into the Hospital Morbidity Information System (*Sistema de informação para Morbilidade Hospitalar*, SIMH), a system developed by the Portuguese Ministry of Health to collect, edit, and group clinical episodes into DRGs or other software applications developed by hospitals. In some institutions, the coding process is documented on paper, and the administrative team then enters the coded data into the SIMH or a specific software program [4].

## Methods

### Study design and participants

We have designed a survey as an ad hoc instrument, based on a literature review and previous work from two focus groups with experienced clinical coders, thereby contributing to item and question definition and covering the main coding processes. A pilot study was conducted to evaluate the clarity, comprehensibility, and face validity of the items. Formal psychometric procedures were not used, as the main goal was to capture descriptive perceptions of coding practices.

The survey was sent in May 2022 online (Google Forms) to a mailing list of long-standing Portuguese clinical coding networks, including national online forums, the mailing list of clinical coding seminars, and additional coder contacts (1400 contacts). Inclusion required experience in clinical coding, confirmed through screening questions; non-coders were excluded. A reminder was sent three weeks after the first email. Participation was voluntary, and informed consent was required. The answers were collected between 10 May 2022 and 20 June 2022.

To increase participation, clinical coders who completed the survey received an index of seminar themes related to clinical coding practice and a list of commonly used acronyms and abbreviations relevant to clinical coding practice.

The invitation to participate contained information about the study and the survey link using Google Forms.

To be included in the study, all participants had to present some experience in clinical coding (current or past). The eligibility of the participants was based on the answers to questions about their work (current or previous) as clinical coders. Eligibility was confirmed through questions about participants’ current or prior involvement in clinical coding, and information on years of experience and, for former coders, the year they ceased coding was also collected.

Before taking part, all participants were informed that their responses would be used for scientific research only and provided informed consent (by accepting a consent statement for the creation of a unique, individual, and anonymous identifier). To assess the possibility of duplicate responses, entries were screened for identical combinations of demographic and professional characteristics. No duplicate responses were identified. However, because responses were collected anonymously, the possibility of undetected duplicates cannot be completely ruled out.

### Survey

The survey included questions to assess the factors influencing the quality of health records, clinical coding, and coded data, as well as their impacts and potential solutions. The survey was developed in Google Forms and included multiple-choice, Likert-type, and short-answer questions. The survey was prepared based on a review of the existing scientific literature on potential problems and barriers to the clinical coding process. The research team discussed which survey items to include. A pilot survey among clinical coders was conducted to assess potential comprehension errors and issues encountered during completion. The full survey instrument is available from the corresponding author upon request.

### Descriptive analysis

A descriptive analysis was conducted to characterise the factors influencing heterogeneity in data quality and the characteristics of the study sample. Continuous variables are presented as median values and 25th and 75th percentiles. Categorical variables are presented in absolute and relative frequency.

R Studio software (RStudio Team, 2022) was used for statistical analysis.

### Inferential analysis

To analyse the association between coders’ personal and professional profiles and their perceptions of various areas of the clinical coding process, a logistic regression analysis was conducted. The full list of variables included in the models is provided in the Supplementary material. Each perception item was modelled as a binary outcome (ratings 1–3 vs 4–5) using logistic regression, with respondents' socio-demographic and professional characteristics included as independent variables. Although rating 3 represents a neutral position, it was grouped with lower ratings to ensure an adequate sample size per category and improve model stability. A stepwise procedure was employed to fit estimated equations, using a backward-forward approach in which explanatory variables are simultaneously added and removed from the model to obtain the best fit.

### Sensitivity analysis

Although all survey respondents had at least some current or former coding experience, recent developments in coding procedures might be overlooked by former coders. Thus, we performed a sensitivity analysis using only those currently performing the coder function. Our results remain virtually unchanged, and the distributions of responses in the full sample and among respondents currently performing clinical coding are presented in Tables S1 and S2 in the Supplementary material, respectively.

Sensitivity analyses were conducted by comparing the distribution of responses in the full sample with. This comparison was descriptive and aimed to assess whether the inclusion of non-active coders influenced overall response patterns.

Sensitivity analyses were also performed for the inferential models. Ordered logit and probit specifications were first estimated using the variables in their original ordinal categorical form, and binary logit and probit models were subsequently re-estimated after collapsing the categories. However, most probit specifications failed to converge under maximum likelihood estimation; therefore, only the results from the binary logit models are reported in Tables S3 to S13 in the Supplementary material.

### Ethical considerations

All phases of this study complied with the Ethical Principles for Medical Research in Human Beings expressed in the Declaration of Helsinki. The Ethics Committee of the Faculty of Medicine, University of Porto, approved the study protocol 11/CEFMUP/2021. Informed consent was obtained from all the respondents.

### STROBE checklist

A STROBE reporting checklist [11] was used when editing this manuscript, included in the Supplementary material.

## Results

### Description of respondents and coding experience

We obtained 162 responses from approximately 1400 potential respondents (i.e. response rate of approximately 11%). The survey respondents were between 25 and 74 years old, with more than half aged 55 or older; median experience was 11 years (p25-p75 3–20 years) and median weekly hours were 8 (p25-p75 4–15 h). Ninety-nine (61%) were female. Most participants coded episodes within their medical field, with 52% of our sample coding across all fields and 68% coding at the same hospital where they practise, with a median of 8 weekly hours (p25-p75: 4–15 h). Most respondents practice in the North region, compared to other regions.

Detailed characteristics are presented in Table 1.

**Table 1:** Sociodemographic and professional characteristics of Portuguese clinical coders participating in a nationwide survey in Portugal (*N* = 162)

**Variables**		**Frequency**	
		**Absolute**	**Relative**
Age group	25–34 years	13	8%
	35–44 years	21	13%
	45–54 years	37	23%
	55–64 years	66	41%
	65–74 years	25	15%
Gender	Female	99	61%
	Male	63	39%
Clinical Field	Medical field	78	48%
	Surgery field	39	24%
	Medical-surgery field	31	19%
	Other	14	9%
Experience in coding (years)	Min	0	
	1 st quartile	3	
	Median	11	
	3rd quartile	20	
	Max	41	
Coder Field	All fields	85	52%
	Medical field	38	23%
	Surgery field	34	21%
	Other	5	3%
Workplace	Same hospital	110	68%
	Same department	40	25%
	Different hospital	34	21%
N. weekly working hours	Min	0,0	
	1 st quartile	4,0	
	Median	8,0	
	3rd quartile	15,0	
	Max	77,0	
Region of the coder	North	67	41%
	Lisbon and Tagus Valley (LVT)	51	31%
	Center	25	15%
	Other	19	12%

### Quality of health records

Regarding the type of health records used during the coding process, almost all respondents reported using electronic records (*N* = 160; 98,8%; IC 95%: 95,6%−99,9%), while 41 (25,3%; IC 95%: 18,8%−32,7%) used paper-based health records. Of this latter group, most (*N* = 39; 24,1%; IC 95%: 17,7%−31,4%) use both records concomitantly. As for the content of health records, almost all use the discharge note (*N* = 161; 99,4%; IC 95%: 96,6%−99,4%), followed by clinical diary (*N* = 151; 93,2%; IC 95%: 88,2%−96,6%), surgical report (*N* = 148; 91,4%; IC 95%: 85,9%−95,2%), anaesthetic report (*N* = 100; 61,7%; IC 95%: 53,8%−69,2%), complementary diagnostic test reports (*N* = 85; 52,5%; IC 95%: 44,5%−60,4%) and nursing records (*N* = 66; 40,7%; IC 95%: 33,1%−48,7%).

**Fig. 1:**
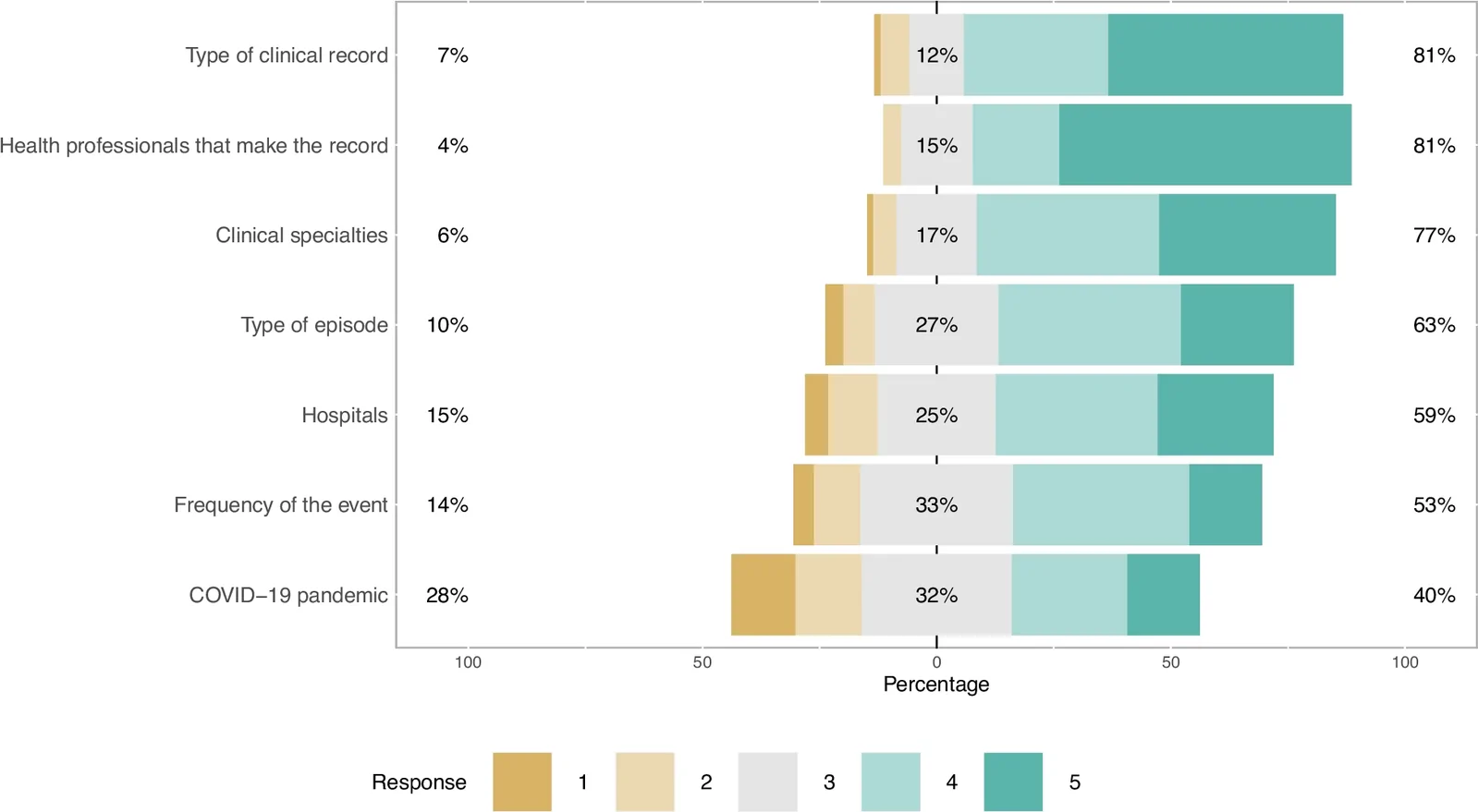
Factors impacting the quality of health records among Portuguese clinical coders in Portugal. Responses are presented as a diverging Likert scale, with ratings of 1–2 displayed to the left of the central axis (lower perceived relevance), rating 3 centred on the axis (neutral), and ratings of 4–5 displayed to the right (higher perceived relevance). The horizontal bars show the percentage of respondents selecting each response category

The respondents' perception of the factors impacting the quality of clinical records (Fig. 1) reveals that most of the listed factors have a high impact, with top-3 being types of clinical records, such as discharge notes or others (80,7%; IC 95%: 73,8%−86,4%), professionals who make the records (80,7%; IC 95%: 73,8%−86,4%) and clinical specialities (76,5%; IC 95%: 69,1%−82,7%). The type of episode being registered, the hospital infrastructure and frequency of the type of episode, patient or pathology (frequent vs. rare event) also was considered factors of impact by the majority of respondents, representing, respectively, 63% (IC 95%: 55%−70,3%), 59,3% (IC 95%: 51,3%−66,8%) and 53,1% (IC 95%: 45,1%−60,9%) of coders.

Respondents were also asked about the impact of COVID-19 on the quality of clinical records, given that it introduced new diagnoses, frequently updated guidelines, and a higher volume of cases, thereby complicating the clinical coding process and requiring ongoing training and adaptation for coders. However, the impact of the COVID-19 pandemic on data quality elicited mixed perceptions among respondents, with no clear consensus on whether it had a positive or negative effect.

**Fig. 2:**
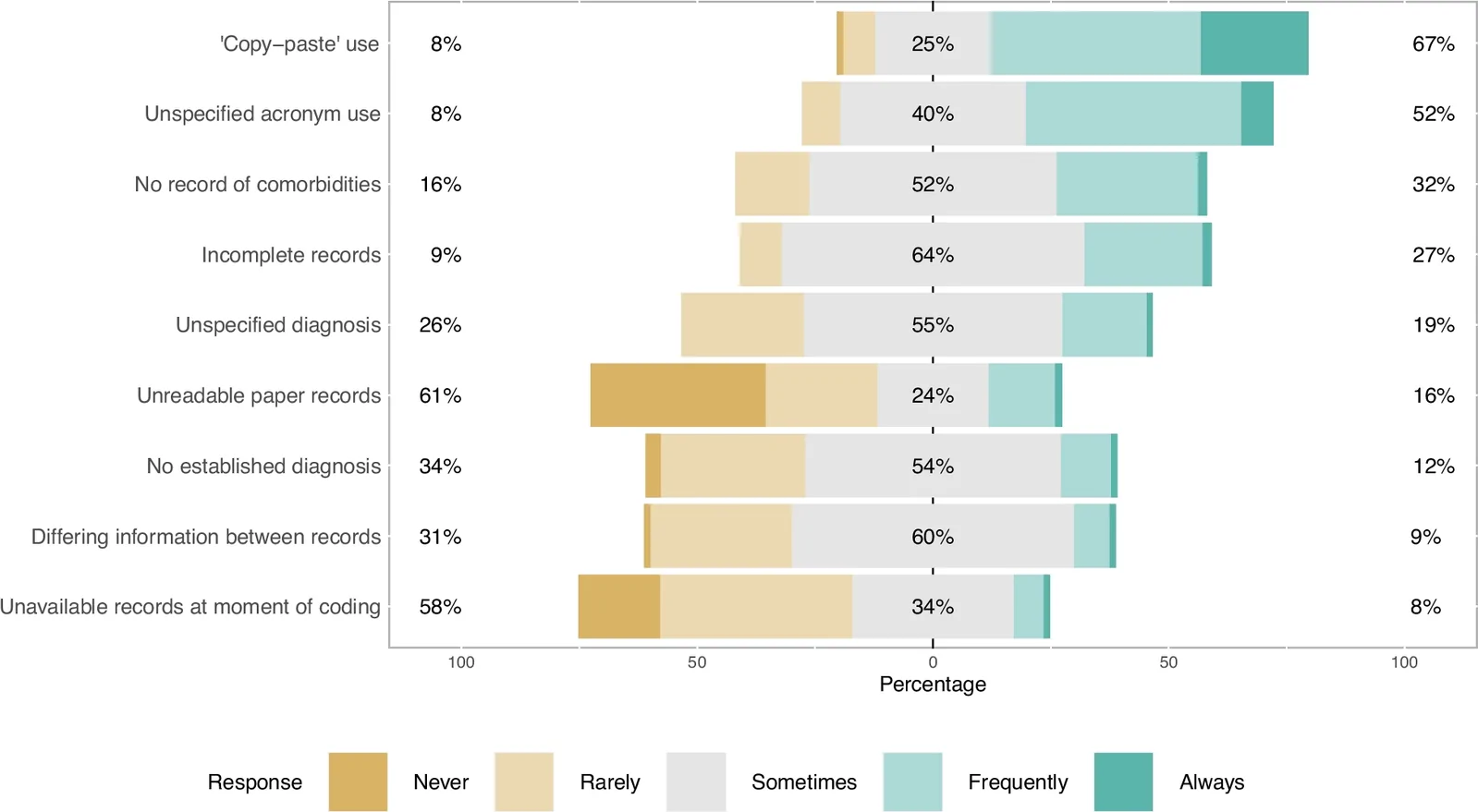
Frequency of documentation problems reported by Portuguese clinical coders participating in a nationwide survey in Portugal. Responses are presented using a five-category frequency scale (Never, Rarely, Sometimes, Frequently, Always), displayed as a diverging stacked bar chart with less frequent responses shown to the left of the central axis and more frequent responses to the right. Percentages represent the proportion of respondents selecting each category

**Fig. 3:**
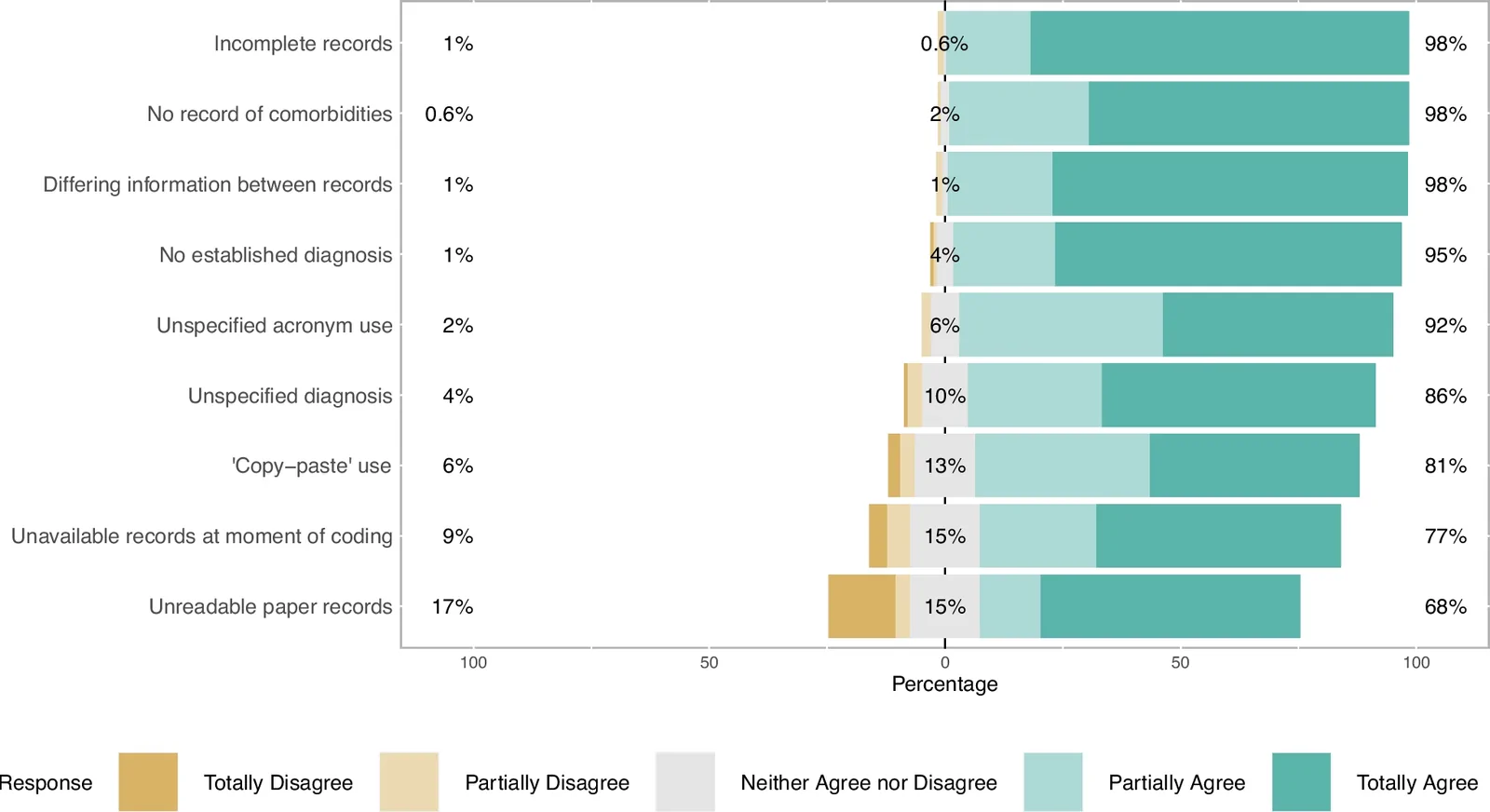
Perceived negative impact of documentation-related factors on coding quality among Portuguese clinical coders participating in a nationwide survey in Portugal. Responses are presented as a diverging Likert scale, with disagreement (totally or partially disagree) displayed to the left of the central axis, neutral responses centred, and agreement (partially or totally agree) displayed to the right. Percentages represent the proportion of respondents selecting each category

The most frequent problems in health records (Fig. 2), according to respondents' perceptions, are the use of “copy-paste” (67,3%; IC 95%: 59,4%−74,3%) and the use of unspecified acronyms (52,5%; IC 95%: 44,5%−60,3%). However, the use of “copy-paste” does not figure among the issues that most negatively impact coding quality (Fig. 3), with 81,5% (IC 95%: 74,5%−87%) of respondents totally or partially agreeing that this factor negatively impacts coding quality. On the other hand, the use of unspecified acronyms was associated with a higher percentage of respondents who totally or partially agreed that the issue negatively affected coding quality (92%; IC 95%: 86,4%−95,5%).

Other problems, such as no record of comorbidities, incomplete records, unspecified or unestablished diagnosis and divergent information between records, were considered occasional occurrences by most respondents (Fig. 2). Despite being non-frequent occurrences, the existence of incomplete records, including comorbidities (98,2%; IC 95%: 94,3%−99,5%) and inconsistent information between records (97,5%; IC 95%: 93,4%−99,2%), was considered by respondents to be the main issue negatively impacting coding quality (Fig. 3). No established or unspecific diagnosis is also an occasional occurrence, with a high percentage of respondents totally or partially agreeing with its negative impact on coding quality, mainly the former, which represents 95,1% (IC 95%: 90,2%−97,7%) and 86,42% (IC 95%: 80%−91,1%), respectively. In contrast, unreadable paper records and the unavailability of records at the time of coding were considered rare or nonexistent. As a result, a higher proportion of respondents totally or partially disagreed with the negative impact of the latter two factors (Fig. 3) compared to the other problems.

**Fig. 4:**
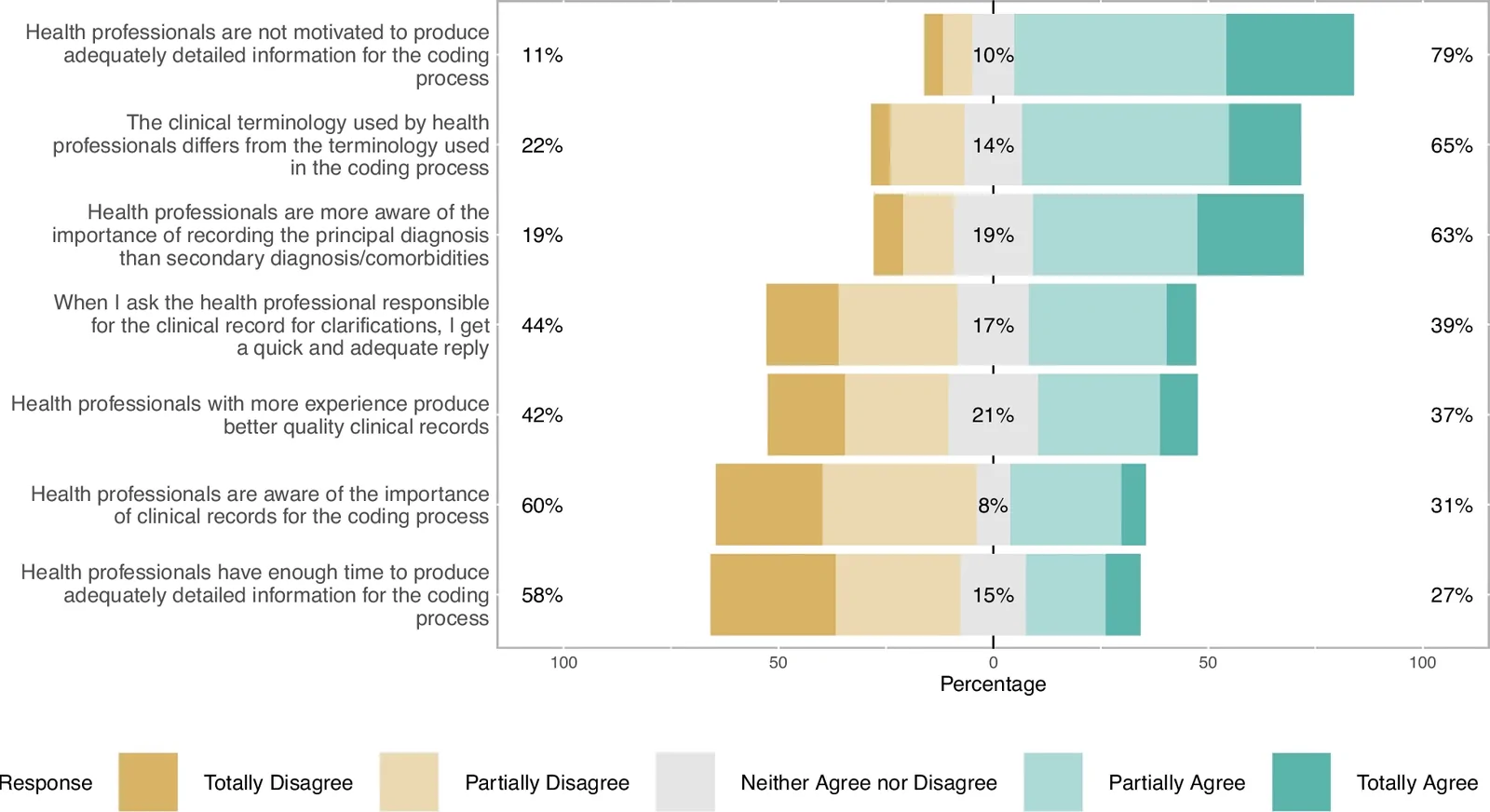
Clinical coders’ perceptions of health professionals’ documentation practices and awareness related to clinical coding in Portugal. Responses are presented as a diverging Likert scale, with disagreement (totally or partially disagree) displayed to the left of the central axis, neutral responses centred, and agreement (partially or totally agree) displayed to the right. Percentages represent the proportion of respondents selecting each category

With regard to health professionals (Fig. 4), the majority of respondents agree that professionals lack the motivation to produce sufficiently detailed information (79%; IC 95%: 71,9%−85%), the terminology used by them differs from that used in the coding process (64,8%; IC 95%: 56,9%−72,1%). They are more aware of the importance of recording the principal diagnosis than the secondary diagnosis or comorbidities (63%; IC 95%: 55%−70,4%). In contrast, most respondents disagree that professionals understand the importance of clinical records in coding (60,5%; IC 95%: 52,5%−68,1%) and that they have enough time to produce adequately detailed information (58%; IC 95%: 50%−65,7%). The ability to reply to clarifications and the relationship between professional experience and the quality of clinical records were points of divergence among respondents (Fig. 4).

### Clinical coding process and quality

**Fig. 5:**
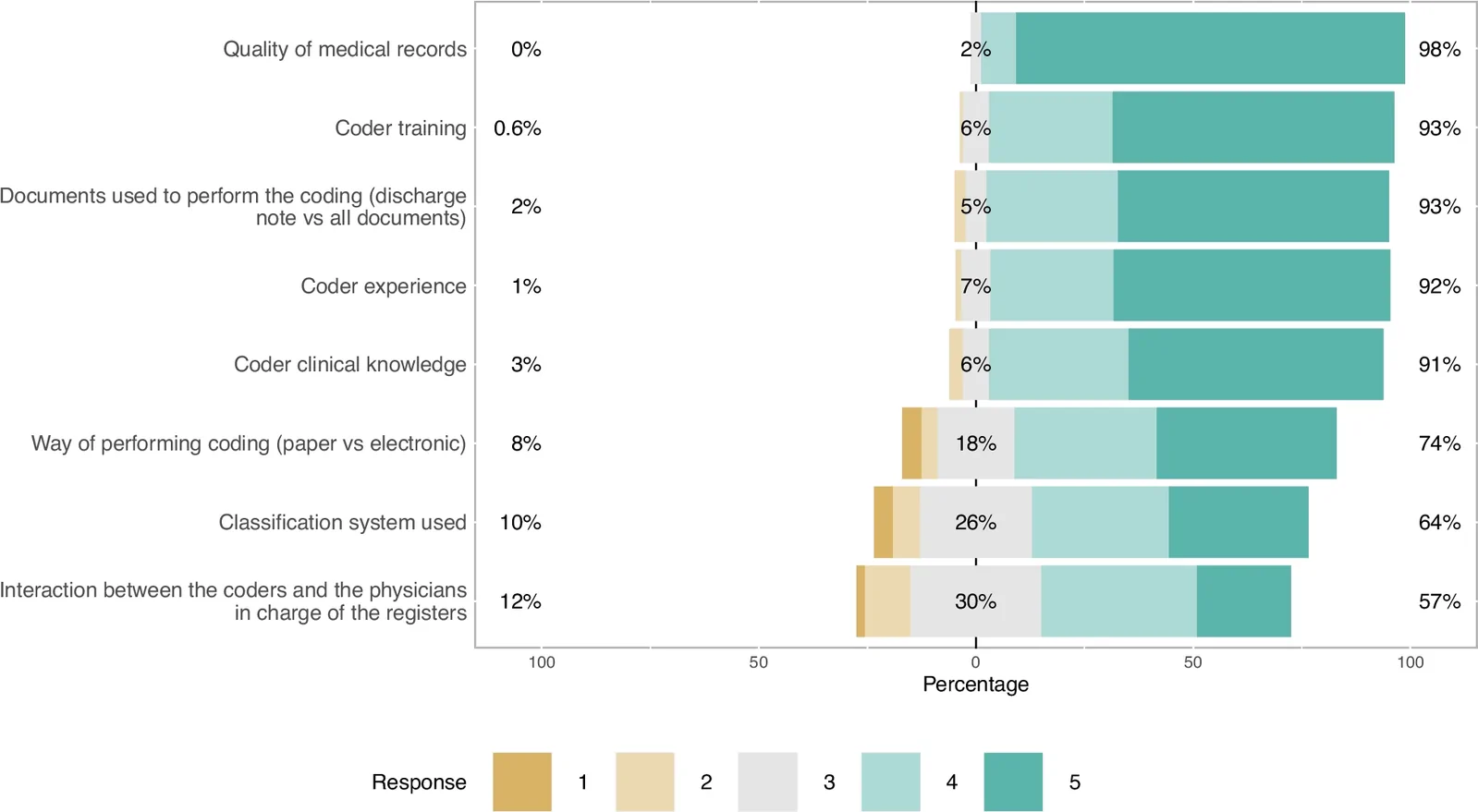
Perceived relevance of factors influencing the quality of clinical coding among Portuguese clinical coders in Portugal. Responses are presented as a diverging Likert scale, with ratings of 1–2 displayed to the left of the central axis (lower perceived relevance), rating 3 centred (neutral), and ratings of 4–5 displayed to the right (higher perceived relevance). Percentages represent the proportion of respondents selecting each category

Respondents' perception of factors impacting coding quality (Fig. 5) revealed consensus on the importance of medical records, with almost all (97,53%; IC 95%: 93,40%−99,21%) assigning a rating of 4 or 5 to this factor. Coder training (93,21%; IC 95%: 87,87%−96,39%), documents used for coding (92,59%; IC 95%: 87,13%−95,94%), coder experience (91,98%; IC 95%: 86,39%−95,48%) and coder clinical knowledge (90,74%; IC 95%: 84,92%−94,55%) were also relevant for most of the respondents. The way of coding (paper-based or electronic), the classification system used, and the interaction between coders and physicians in charge of registers were also perceived as necessary for most respondents; however, opinions on these factors showed greater variability along the rating scale, with a higher proportion of ratings of 1, 2, or 3 compared to the other factors impacting coding quality.

**Fig. 6:**
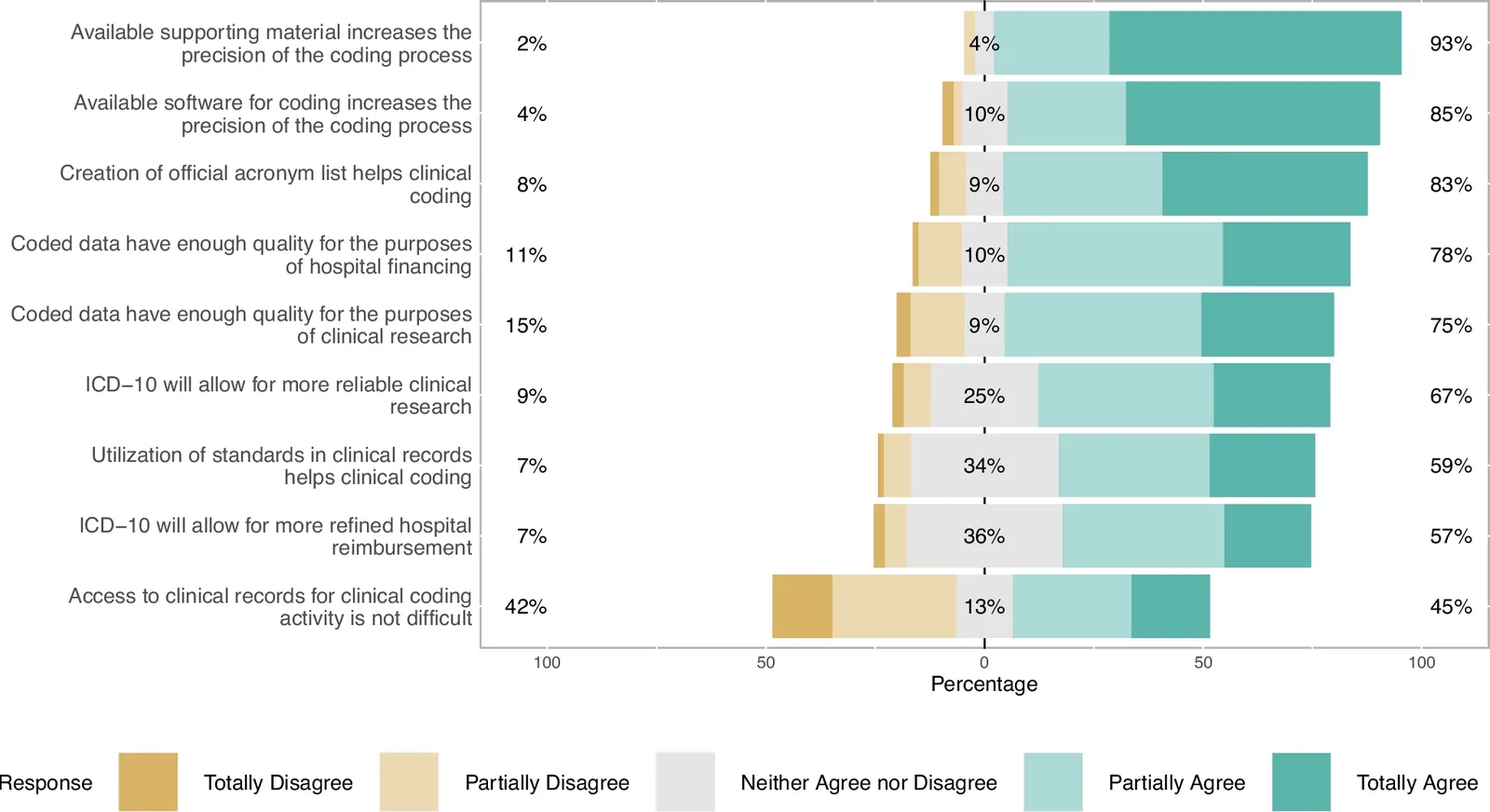
Clinical coders’ perceptions of resources, standards, and system factors supporting clinical coding in Portugal. Responses are presented as a diverging Likert scale, with disagreement (totally or partially disagree) displayed to the left of the central axis, neutral responses centred, and agreement (partially or totally agree) displayed to the right. Percentages represent the proportion of respondents selecting each category

Regarding the factors impacting clinical coding itself (Fig. 6), there was high agreement from respondents on the availability of support materials (93,2%; IC 95%: 87,9%−96,4%) and coding software (85,2%; IC 95%: 78,6%−90,1%) to increase the precision of the coding process, along with the creation of an acronym list to help clinical coding (83,3%; IC 95%: 76,5%−88,6%). Most respondents also supported the idea that coded data has sufficient quality for the purposes of hospital funding (78,4%; IC 95%: 71,1%−84,3%) and clinical research (75,3%; IC 95%: 67,8%−81,6%); ICD-10-CM/PCS will allow more reliable clinical research (66,7%; IC 95%: 58,8%−73,8%), the use of standards in clinical records helps clinical coding (58,6%; IC 95%: 50,6%−66,2%) and ICD-10-CM/PCS will allow more rigorous hospital reimbursement (56,8%; IC 95%: 48,8%−64,5%). Respondents differed in their perceptions of whether access to clinical records for clinical coding is difficult, with a balanced division between those who disagree (42%; IC 95%: 34,4%−50%) and those who agree (45,1%; IC 95%: 37,3%−53,1%). The divergence in respondents' perceptions was also observed when asked to classify the difficulty level of defining a principal diagnosis, coding procedures, priority diagnoses relative to the maximum number of codes, and identifying secondary diagnoses or comorbidities (Fig. 6).

**Fig. 7:**
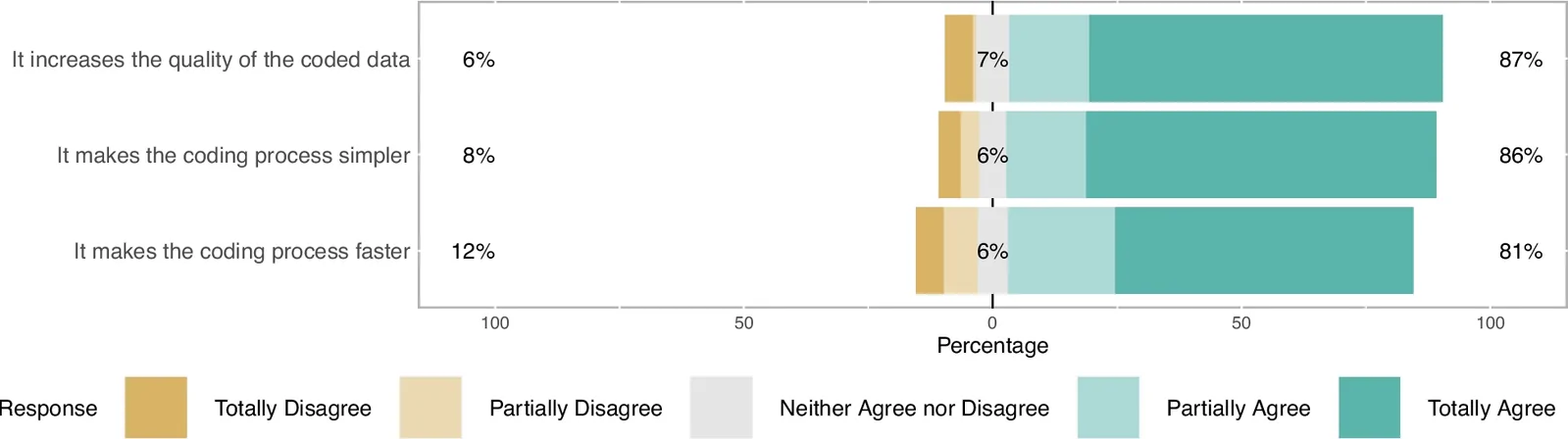
Clinical coders’ perceptions of the benefits of electronic coding in Portugal. Responses are presented as a diverging Likert scale, with disagreement (totally or partially disagree) displayed to the left of the central axis, neutral responses centred, and agreement (partially or totally agree) displayed to the right. Percentages represent the proportion of respondents selecting each category

**Fig. 8:**
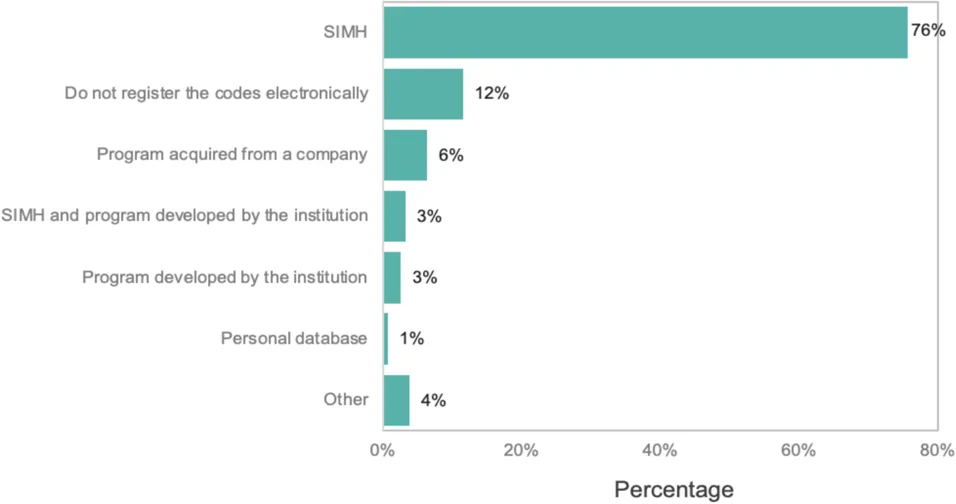
Software used for electronic clinical coding among Portuguese clinical coders in. Percentages represent the proportion of respondents who reported using each software

Most respondents agree that electronic coding (Fig. 7) increases the quality of coded data (87,1%; IC 95%: 80,9%−91,8%) and makes the coding process simpler (86,2%; IC 95%: 80,2%−91,3%) and faster (81,5%; IC 95%: 74,6%−87,1%). When asked about the software used for electronic coding (Fig. 8), most respondents (75,9%; IC 95%: 68,6%−82,3%) reported using only the SIMH.

**Fig. 9:**
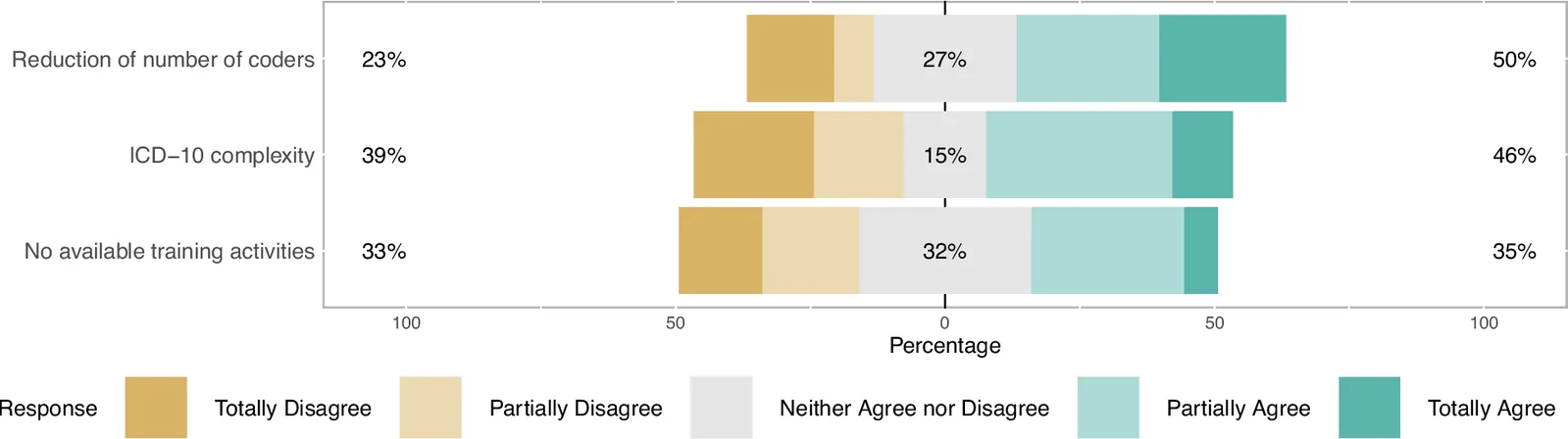
Clinical coders’ perceptions of factors contributing to delays in clinical coding in. Responses are presented as a diverging Likert scale, with disagreement (totally or partially disagree) displayed to the left of the central axis, neutral responses centred, and agreement (partially or totally agree) displayed to the right. Percentages represent the proportion of respondents selecting each category

Regarding the delay in coding, 34% of respondents reported no delay (*N* = 55; IC 95%: 26,7%−41,8%) and 31,5% (*N* = 51; IC 95%: 24,4%−39,2%) reported not knowing the delay, 24,7% (*N* = 40; IC 95%: 18,3%−32,1%) reported a delay between 2 and 5 months, and 9,9% (*N* = 16; IC 95%: 5,8%−15,5%) reported more than 6 months of delay. When asked to answer to what extent they agree (or disagree) with factors related to delays in coding (Fig. 9), 50% (IC 95%: 42,4%−57,6%) of respondents agreed with the factor related to the reduction in the number of coders. At the same time, the complexity of ICD-10 and the unavailability of training activities were factors contributing to greater divergence among respondents.

**Fig. 10:**
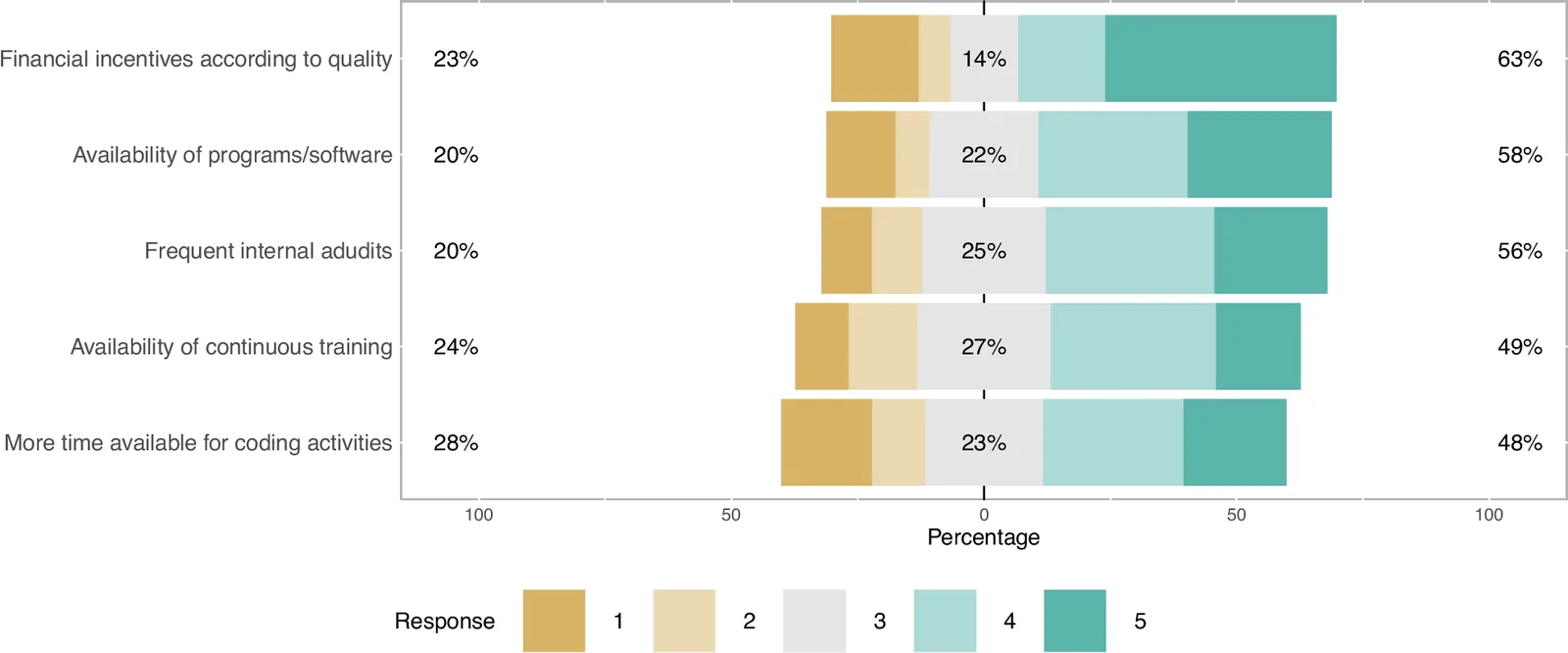
Priorities identified by Portuguese clinical coders for improving hospital morbidity data quality in Portugal. Responses are presented as a diverging Likert scale, with ratings of 1–2 displayed to the left of the central axis (lower priority), rating 3 centred (neutral), and ratings of 4–5 displayed to the right (higher priority). Percentages represent the proportion of respondents selecting each category

Regarding the priority measures for increasing data quality (Fig. 10), most respondents attributed high priority to financial incentives according to quality (63%; IC 95%: 55%−70,3%), the availability of programs/software (58%; IC 95%: 50%−65,7%) and frequent internal audits (55,6%; IC 95%: 47,6%−63,3%). Regarding auditing, only 37,7% (*N* = 61; IC 95%: 30,2%−45,6%) of the coders’ workplaces provide internal and external auditing, while 21% (*N* = 34; IC 95%: 15%−28,1%) reported only internal auditing and 5,6% (*N* = 9; IC 95%: 2,6%−10,3%) only external auditing. Many respondents (*N* = 50, 30,9%; IC 95%: 23,9%−38,6%) reported being unaware of auditing, and 4,9% (*N* = 8; IC 95%: 2,2%−9,5%) reported no auditing provided by the workplace.

### Inferential analysis

Regarding the results of binary logit regressions, three patterns emerged across the models: (i) paper-based or mixed coding workflows were associated with less favourable perceptions of coding quality and precision; (ii) coders working in the same hospital or department reported fewer problems with unavailable or inconsistent documentation; and (iii) the use of structured clinical summaries was associated with fewer reports of unspecified or missing diagnoses. It should be kept in mind, however, that the regression findings should be regarded as exploratory, due to low cell counts in several categories and quasi-complete separation in some models. More details about the full outputs may be found in Tables S4 to S13 in the Supplementary material.

## Discussion

According to participants in this nationwide Portuguese survey, clinical coders indicate that the types of clinical records (81%), the professionals who create them (81%), and clinical specialities (77%) are the top factors influencing the quality of clinical records. Furthermore, they believe that health professionals are not motivated by the importance of carefully, completely and comprehensively producing health records (79%), nor are they aware of the importance of clinical records for the coding process (58%), which could explain heterogeneities in quality by speciality and by the type of clinical record. They consider that health professionals do not have enough time to record information in detail for coding (58%). These various factors can have repercussions on the errors in clinical records that the clinical coders reported as most frequent, namely the use of copy-paste, unspecified acronyms or incomplete records. In addition to the high frequency, most participants consider these factors negatively impact the coding process. Potential factors affecting the quality of clinical records were identified in a 2017 qualitative study through focus groups with clinical coders [3]. These findings are consistent with previous qualitative studies conducted in Portugal using focus groups, in which clinical coders identified a lack of information in the health record, unclear documentation, variability in health records, and a lack of solutions to these problems. In the paper, some suggestions to overcome these problems are discussed, namely the standardisation of the structure and content of clinical records, the development of a standard nomenclature, and the existence of audits. Using a broader group through this survey, clinical coders report the same difficulties and problems, which leads us to believe that the strategies have not yet been sufficiently established.

This study adds to the literature by providing a nationwide quantitative survey of medical coders’ perceptions in Portugal, allowing not only the identification but also the prioritisation of key factors affecting the quality of hospital morbidity data. It highlights the differential impact of common issues such as incomplete records, unspecified acronyms, and “copy-paste” practices. Furthermore, it extends previous work by linking these challenges to concrete, system-level interventions and aligning these findings with data quality frameworks.

These difficulties are not exclusive to Portugal. In Canada, coders consider clinical records the main barriers, proposing educational sessions for health professionals to alert them to write understandable clinical records appropriate to the coding process [44, 45].

Regarding incomplete records, participants reported that health professionals are more aware of the importance of recording the primary diagnosis than the secondary diagnosis or comorbidities (63%). The non-coding of secondary diagnoses and comorbidities is a well-known issue with implications for hospital funding and data quality that can be used for epidemiological or research purposes [16, 27, 38, 42].

When there is any doubt about clinical records due to incompleteness or incongruity, health professionals are often contacted to clarify matters for the clinical coders. The participants differed in their opinions about this process: 39% agreed with the statement that they received a quick and adequate response, 44% disagreed, and 17% neither agreed nor disagreed. This variability likely reflects organisational heterogeneity and differences in communication workflows, with some institutions relying mostly on informal contacts. A recent process reference model for the Portuguese context proposes, amongst a comprehensive set of recommendations, the definition of unified norms for timeliness coder-physician queries [15]. Moreover, empirical evidence points to institutional differences in clinical documentation practices in Portugal, including instances in which discharge notes are written by physicians who were not directly responsible for the patient [3]. Other potential explanations include high clinician workload and time constraints, which may vary by hospital size and complexity. In our study, 58% of respondents reported that clinicians have insufficient time for detailed documentation, which likely affects timely response to coding queries.

In addition to the quality of medical records, the factor with the most significant agreement was the documents used in the coding process, and participants also recognised some characteristics of the coding doctor that had a significant impact on coding quality. Both coder training, coder experience, and coder clinical knowledge were rated 4 or 5 in relevance by more than 90% of participants. These findings suggest that documentation quality reflects not only technical limitations but also organisational and behavioural factors, such as time constraints and variability in clinical practice.

Participants also consider that some tools and strategies can contribute to the quality of coded data, namely the availability of supporting materials, coding software and lists of acronyms that support the clinical coder.

The transition from ICD-9-CM to ICD-10-CM/PCS has also posed slightly greater challenges for clinical coders, particularly regarding training and adaptation. In Portugal, this transition began in 2016 and was marked by heterogeneities across hospitals [30]. Despite these issues, many coders have preferred the new classification system [2]. A key issue for a successful transition to ICD-10-CM/PCS, concerning the improvement of clinical coding quality, is providing adequate training to coders [18, 21]. Although it was less relevant than training, the coding system was also rated 4 or 5 in coding quality by more than half of the participants. More than half of the participants consider that changing the ICD-10-CM/PCS classification system allows for more reliable clinical research (67%) and more refined hospital reimbursement (57%). The differences between the ICD-9-CM and ICD-10-CM/PCS classification systems, as well as the difficulties encountered during the transition, were also evaluated in this study, and the conclusions have already been published [25]. In that article, only answers from clinical coders with experience with ICD-9-CM and ICD-10-CM/PCS were included. Participants noted that the greater complexity of ICD-10-CM/PCS compared to ICD-9-CM led to a temporary decrease in coding quality as coders struggled to adapt to the new system, increasing coding time and affecting productivity [25].

The impact of the COVID-19 pandemic on the quality of records was relatively balanced among respondents, with some attributing a significant effect to it. Regarding coding quality, almost all respondents reported incomplete records and inconsistent information as significant issues (98%). Even though all these problems were identified during the coding process, which allows for the development of strategies to improve, it is worth noting that 78% of participants consider that the coded data is of sufficient quality for hospital financing, and 75% for clinical research. The quality of health data deserves special attention because of the implications it could have for hospital funding and its reuse.

Multivariable models showed signs of instability, with some odds ratios exceeding 100 and inconsistent directions, reflecting sparse data across several categories. This limitation is expected in surveys involving heterogeneous groups and highlights the need to interpret multivariable findings with caution.

Most issues and recommendations identified by Portuguese coders can be mapped to well-established Data Quality Assessment (DQA) frameworks. Table 2 presents the mapping of the issues and recommendations to the related dimensions proposed in selected frameworks.

**Table 2:** Mapping of survey-identified data quality issues and recommendations to established data quality assessment (DQA) frameworks

**Issue/Recommendation**	Kahn et al. [20]** Framework**	Weiskopf et al. [47]** Framework**	Wang & Strong [46]** Framework**	ISO 25012 [19] Data Quality Model
Incomplete, inconsistent, or missing data in health records	Completeness	Completeness	Accuracy (Intrinsic Data Quality, Completeness (Contextual Data Quality)	Completeness, Accuracy, Consistency
Unspecified acronyms, “copy-paste,” divergent documentation	Conformance (e.g., standards and internal rules)	Representational Consistency	Interpretability, Consistency (Representational Data Quality)	Understandability, Accuracy, Compliance
Health professionals lack awareness/time to record accurately	Plausibility	Trustworthiness	Credibility, Believability (Intrinsic Data Quality)	Credibility, Accuracy
Use of electronic systems and software tools	Conformance (e.g., to data formats and standards)	Accessibility/Reusability	Ease of access, security (Accessibility Data Quality)	Availability, Accessibility, Portability, Recoverability, Traceability
Internal/external audits and financial incentives for quality	Conformance, Plausibility	Validation/Verification	Reliability, Value-added (Intrinsic and Contextual Quality)	Compliance, Integrity, Auditability, Credibility
Transition to ICD-10-CM/PCS	Conformance, Completeness	Correctness, Granularity	Relevancy, Timeliness, Value-added (Contextual Data Quality)	Compliance, Completeness, Accuracy, Currentness
Collaboration between coders and clinicians	Plausibility	Consistency, Concordance	Interpretability, Understandability (Representational Quality)	Consistency, Accuracy, Traceability, Understandability

This mapping places existing issues within internationally recognised standards and demonstrates that our findings intersect several complementary DQA frameworks, comprising micro-level data dimensions (e.g., accuracy, completeness, and consistency) and macro-level data governance dimensions (e.g., the ISO 25012 [19] model). To address these issues, it is paramount to define and maintain unified hospital-level standards, policies, rules, and guidelines for coding, training, and auditing, and to manage updates to terminology and reference materials. A unified national web portal for policy dissemination and KPI monitoring can further help to achieve these goals and implement data governance and DQA initiatives across hospitals. Examples of key actionable DQA steps tailored to the Portuguese scenario and derived from our findings are presented in Table 3.

**Table 3:** Proposed data quality improvement actions, indicators, and targets for hospital morbidity data in Portugal

DQA Area	KPI/metric	Target	Owner	Cadence
*Documentation Quality*	Proportion of records with complete documentation fields Proportion of records returned by medical coders to physicians with requests for more information or documentation to support code assignment	> 95% < 5%	Hospital Department or Services responsible for the related documentation	Monthly
*Acronym and Terminology Governance*	Proportion of records with unrecognised or non-standard acronyms	< 5%	Clinical coding office managers	Monthly
*Copy-Paste Control*	Proportion of records with duplicated content	< 10%	Clinical coding office managers	Monthly
*Comorbidity Reporting*	Median number of comorbidity and complications codes per base DRG	Deviance of up to 5% of national median (benchmark)	Clinical coding office managers	Monthly
*Collaboration between coders & clinicians*	Proportion of queries resolved within 7 days	> 99%	Hospital Department or Services responsible for the related documentation	Monthly
*Coders training and feedback*	Internal and external audit accurace rate Coder productivity: median number of admissions coded per day	> 95% Deviance of up to 5% of national median defined by hospital type (benchmark)	Hospital Administration, Clinical coding office managers	Quarterly
*Internal/external audits and financial incentives for quality*	Proportion of episodes rejected before entering the national morbidity database, following internal audit	< 5%	Hospital Administration, clinical coding office managers	Quarterly

The target values were based on published benchmarks from coding audits and administrative evaluations, in which coding accuracy and completeness above 90% and high physician response rates to queries (e.g., 97%) are considered operational standards in health information management [13]. A systematic review further indicates that hospitals maintaining coding accuracy above 90% are associated with improved case-mix adjustment, fairer benchmarking, and more accurate mortality reporting [14]. Empirical findings from the Portuguese context informed prioritisation, namely heterogeneity in comorbidity reporting [42], and previously identified coding barriers (e.g., frequent use of acronyms and copy-paste practices, lack of incentives for quality) [3, 4]. Monthly monitoring was defined for most KPIs to align with operational requirements in public Portuguese hospitals, as episodes must be coded by the seventh day of the following month, requiring timely and pragmatic evaluation processes [41].

### Strengths and limitations

This study obtained 162 responses from approximately 11% of potential responders. This low participation rate may have introduced non-response bias, as a formal non-response analysis was not possible due to the unavailability of data on non-responders. Nevertheless, the sample includes experienced professionals (with a median of 11 years of clinical coding), providing sufficient depth to address the research question. Although it would have been beneficial to obtain a higher number of respondents from each administrative region, including assessing whether organisational factors compromise the quality of coded data, these numbers reflect the regional distribution of physicians.

Regarding inferential analysis, many categories had few observations, leading some regression models to exhibit quasi-complete separation and, consequently, unstable effect estimates. For this reason, regression findings are interpreted cautiously and presented as exploratory.

Additionally, the survey instrument was specifically developed for this study and was not submitted to formal psychometric validation. Although its content was supported by a literature review, expert contributions, and a pilot test, this should be considered, as it might limit comparability with other studies.

### Implications

The primary issues affecting the quality of health records include the prevalence of "copy-paste" practices, the use of unspecified acronyms, incomplete documentation, and divergent information across records. While Electronic Medical Records (EMRs) can improve data quality, issues such as incomplete or incorrect data entry can lead to significant underestimation of quality indicators [9]. The perceived quality of patient data is closely linked to the ease of use and alignment of EMRs with daily hospital routines [22]. However, EMRs have also introduced new challenges, such as the "copy and paste" functionality, which can propagate errors throughout patient records, underscoring the need for well-designed EMR systems [3]. High work volume and complexity are also significant challenges, with evidence showing that smaller hospitals generally achieve better coding accuracy than larger hospitals due to the high complexity and volume of cases of the latter [23, 29]. These problems can be addressed by implementing documentation guidelines and by training healthcare professionals on the importance of detailed, accurate record-keeping. Additionally, employing documentation improvement specialists and computer-assisted coding tools can enhance the quality of clinical records. Encouraging better communication between healthcare providers and coders can also help mitigate discrepancies and ensure more comprehensive, accurate documentation. The coding process faces challenges, including incomplete and nonspecific documentation, variability in documentation practices across specialities, and communication gaps between coders and healthcare providers. In fact, recent studies support these findings, showing that shortcomings in documentation are often linked not only to clinician workload but also to structural issues such as EMR usability, lack of interoperability, and inconsistent adoption of digital tools [48–50]. To address these issues, standardising documentation practices and adopting electronic coding systems can significantly improve the consistency and accuracy of the coding process [1, 17]. Providing continuous training for coders, implementing clear coding policies, and fostering collaboration between coders and healthcare professionals are essential steps to improve the coding process. Additionally, the availability of support materials and coding software can improve coding precision and efficiency.

The quality of coded data is compromised by issues like incomplete documentation, lack of specificity, and the use of non-standard acronyms. To solve these problems, healthcare organisations can focus on improving the overall quality of health records. Coding quality can be improved by engaging coders in various role behaviours, providing better career opportunities, increasing staff numbers, allowing more time for coding tasks, fostering more significant interaction between coders and other medical staff, providing financial incentives for high-quality documentation, and integrating advanced electronic health record systems that facilitate accurate and comprehensive data entry [39]. Audits are also part of the solution for addressing problems with health records and ensuring the quality of clinical coding. The development of tools to assist with coding and regular auditing practices is essential for maintaining high standards [3, 4], with particular emphasis on attributes such as dataset accuracy and completeness [26]. Ensuring that coders have access to complete and legible records and providing ongoing education about the importance of detailed coding will also contribute to higher-quality coded data.

This study has an important impact on coding and epidemiological profiles, as well as on economic impact and policy design [28, 31, 33, 34, 36]. Hospital morbidity data is crucial for epidemiological profiling and disease burden measurement, providing valuable information on disease prevalence and incidence. High-quality morbidity data enhance the accuracy and reliability of burden-of-disease estimates and cost-of-illness analyses [32, 35]. However, researchers must remain cautious of potential biases, such as incomplete data, when utilising administrative databases. In fact, accurate hospital morbidity data can also inform public health practices and support better decision-making in healthcare planning and policy development [36, 37].

The findings of this survey can also have significant implications for the European Health Data Space (EHDS). The EHDS aims to leverage shared European data infrastructures to advance public health, research, and innovation by ensuring the quality and interoperability of health data [12]. Efforts to improve data quality and semantic interoperability are ongoing to support the European Union and national authorities in addressing data quality challenges and ensuring the reliability of health data for secondary use within the EHDS.

Other European countries can draw valuable lessons from the challenges and solutions identified in this study. Standardising documentation practices, improving coder training, and adopting advanced electronic health record systems are essential steps towards achieving better epidemiological profiles and planning health systems and health care. Moreover, the insights from this survey can guide policymaking and the implementation of best practices across Europe, ultimately improving the quality and reliability of health data for better patient outcomes and public health research.

In conclusion, heterogeneity in morbidity data quality in Portugal arises from multiple factors identified by clinical coders, highlighting variability in the type of record used for coding, differences in data quality between specialities and a lack of awareness of the importance of producing accurate health records. Challenges at the hospital level persist, namely insufficient time to produce complete documentation and adaptation to ICD-10-CM/PCS. Nevertheless, current efforts to address hospital morbidity are making a difference for health planning and health systems. Coders’ training, supporting materials, and software have been recognised as potential solutions, even though these practices are currently inconsistent across hospitals. Moreover, addressing data quality heterogeneity will require systematic and harmonised interventions, including the standardisation of medical records, increased collaboration between coders and professionals responsible for documentation, and promotion of education regarding the quality of patient documentation.

## Supplementary Information

Supplementary Material 1.

## Data Availability

The dataset generated and/or analysed during the current study is not publicly available due to privacy and confidentiality restrictions, but is available from the corresponding author on reasonable request.
