# Supplementary material for: Why is the data quality of hospital morbidity so heterogeneous? A nationwide survey of Portuguese clinical coders
Source: Arch Public Health. 2026 Jul 7;84:205. doi: 10.1186/s13690-026-01985-1 (PMC13576173; doi:10.1186/s13690-026-01985-1)
Supplement: Supplementary file 1 [file 13690_2026_1985_MOESM1_ESM.docx]

**SUPPLEMENTARY MATERIAL**

1. **Sensitivity analysis**

Table S1. Distribution of responses with the full sample.

|  | Response group | | | | | | |
| --- | --- | --- | --- | --- | --- | --- | --- |
| Item | 1 | 2 | 3 | 4 | 5 | 6 | 7 |
| More time available for coding activities | 18 | 10 | 23 | 28 | 20 |  |  |
| Availability of continuous training | 10 | 14 | 27 | 33 | 17 |  |  |
| Availability of programs/software | 14 | 7 | 22 | 30 | 28 |  |  |
| Financial incentives according to quality | 17 | 6 | 14 | 17 | 46 |  |  |
| Frequent internal audits | 10 | 10 | 25 | 33 | 22 |  |  |
| Hospitals | 5 | 10 | 25 | 35 | 25 |  |  |
| Clinical specialties | 1 | 5 | 17 | 39 | 38 |  |  |
| Health professionals that make the record | 0 | 4 | 15 | 19 | 62 |  |  |
| Type of clinical record | 1 | 6 | 12 | 31 | 50 |  |  |
| Type of episode | 4 | 7 | 27 | 39 | 24 |  |  |
| Frequency of the event | 4 | 10 | 33 | 38 | 15 |  |  |
| COVID-19 pandemic | 14 | 14 | 32 | 25 | 15 |  |  |
| Principal diagnosis definition | 4 | 27 | 30 | 28 | 12 |  |  |
| Identification of secondary diagnosis and comorbidities | 4 | 28 | 36 | 27 | 4 |  |  |
| Definition of diagnostic priorities (in relation to the max. number of codes) | 7 | 22 | 38 | 28 | 4 |  |  |
| Coding of procedures | 5 | 25 | 35 | 27 | 9 |  |  |
| Quality of medical records | 0 | 0 | 2 | 8 | 90 |  |  |
| Interaction between the coders and the physicians in charge of the registers | 2 | 10 | 30 | 36 | 22 |  |  |
| Classification system used | 4 | 6 | 26 | 31 | 32 |  |  |
| Documents used to perform the coding (discharge note vs all documents) | 0 | 2 | 5 | 30 | 62 |  |  |
| Way of performing coding (paper vs electronic) | 4 | 4 | 18 | 33 | 41 |  |  |
| Coder training | 0 | 1 | 6 | 28 | 65 |  |  |
| Coder experience | 0 | 1 | 7 | 28 | 64 |  |  |
| Coder clinical knowledge | 0 | 3 | 6 | 32 | 59 |  |  |
| Utilization of standards in clinical records helps clinical coding | 1 | 6 | 34 | 35 | 24 |  |  |
| Creation of official acronym list helps clinical coding | 2 | 6 | 9 | 36 | 47 |  |  |
| Access to clinical records for clinical coding activity is not difficult | 14 | 28 | 13 | 27 | 18 |  |  |
| Available supporting material increases the precision of the coding process | 0 | 2 | 4 | 27 | 67 |  |  |
| Available software for coding increases the precision of the coding process | 2 | 2 | 10 | 27 | 58 |  |  |
| Coded data have enough quality for the purposes of hospital financing | 1 | 10 | 10 | 49 | 29 |  |  |
| Coded data have enough quality for the purposes of clinical research | 3 | 12 | 9 | 45 | 30 |  |  |
| ICD-10 will allow for more refined hospital reimbursement | 2 | 5 | 36 | 37 | 20 |  |  |
| ICD-10 will allow for more reliable clinical research | 2 | 6 | 25 | 40 | 27 |  |  |
| Utilization of standards in clinical records helps clinical coding | 1 | 6 | 34 | 35 | 24 |  |  |
| Creation of official acronym list helps clinical coding | 2 | 6 | 9 | 36 | 47 |  |  |
| Access to clinical records for clinical coding activity is not difficult | 14 | 28 | 13 | 27 | 18 |  |  |
| Available supporting material increases the precision of the coding process | 0 | 2 | 4 | 27 | 67 |  |  |
| Available software for coding increases the precision of the coding process | 2 | 2 | 10 | 27 | 58 |  |  |
| Coded data have enough quality for the purposes of hospital financing | 1 | 10 | 10 | 49 | 29 |  |  |
| Coded data have enough quality for the purposes of clinical research | 3 | 12 | 9 | 45 | 30 |  |  |
| ICD-10 complexity | 22 | 17 | 15 | 35 | 11 |  |  |
| Reduction of number of coders | 16 | 7 | 27 | 27 | 23 |  |  |
| No available training activities | 15 | 18 | 32 | 28 | 6 |  |  |
| Evolution of medical record quality since 2018 | 22 | 43 | 7 | 20 | 6 | 2 |  |
| Incomplete records | 0 | 9 | 64 | 25 | 2 |  |  |
| Differing information between records | 1 | 30 | 60 | 8 | 1 |  |  |
| No established diagnosis | 3 | 31 | 54 | 11 | 1 |  |  |
| No record of comorbidities | 0 | 16 | 53 | 30 | 2 |  |  |
| Unspecified acronym use | 0 | 8 | 40 | 46 | 7 |  |  |
| 'Copy-paste' use | 1 | 7 | 25 | 44 | 23 |  |  |
| Unreadable paper records | 37 | 24 | 24 | 14 | 1 |  |  |
| Unspecified diagnosis | 0 | 26 | 55 | 18 | 1 |  |  |
| Unavailable records at moment of coding | 17 | 41 | 34 | 6 | 1 |  |  |
| Health professionals are aware of the importance of clinical records for the coding process | 25 | 36 | 8 | 26 | 6 |  |  |
| When I ask the health professional responsible for the clinical record for clarifications, I get a quick and adequate reply | 17 | 28 | 17 | 32 | 7 |  |  |
| Health professionals are not motivated to produce adequately detailed information for the coding process | 4 | 7 | 10 | 49 | 30 |  |  |
| Health professionals have enough time to produce adequately detailed information for the coding process | 29 | 29 | 15 | 19 | 8 |  |  |
| The clinical terminology used by health professionals differs from the terminology used in the coding process | 4 | 17 | 14 | 48 | 17 |  |  |
| Health professionals with more experience produce better quality clinical records | 18 | 24 | 21 | 28 | 9 |  |  |
| Health professionals are more aware of the importance of recording the principal diagnosis than secondary diagnosis/comorbidities | 7 | 12 | 19 | 38 | 25 |  |  |
| Incomplete records | 0 | 1 | 1 | 18 | 80 |  |  |
| Differing information between records | 0 | 1 | 1 | 22 | 75 |  |  |
| No established diagnosis | 1 | 1 | 4 | 22 | 73 |  |  |
| No record of comorbidities | 0 | 1 | 2 | 30 | 68 |  |  |
| Unspecified acronym use | 0 | 2 | 6 | 43 | 49 |  |  |
| 'Copy-paste' use | 2 | 3 | 13 | 37 | 44 |  |  |
| Unreadable paper records | 14 | 3 | 15 | 13 | 55 |  |  |
| Unspecified diagnosis | 1 | 3 | 10 | 28 | 58 |  |  |
| Unavailable records at moment of coding | 4 | 5 | 15 | 25 | 52 |  |  |
| How often do you receive feedback on the quality of your data? | 30 | 7 | 22 | 12 | 9 | 17 | 3 |
| How often do you meet with an auditor to assess the quality of your data? | 39 | 10 | 20 | 7 | 11 | 9 | 3 |
| How often do you consider it relevant to have continuing education? | 1 | 32 | 4 | 18 | 13 | 26 | 5 |
| It makes the coding process faster | 6 | 7 | 6 | 22 | 60 |  |  |
| It makes the coding process simpler | 4 | 4 | 6 | 16 | 70 |  |  |
| It increases the quality of the coded data | 6 | 1 | 7 | 16 | 71 |  |  |

Table S2. Distribution of responses for the subgroup of respondents currently performing clinical coding.

|  | Response group | | | | | | |
| --- | --- | --- | --- | --- | --- | --- | --- |
| Item | 1 | 2 | 3 | 4 | 5 | 6 | 7 |
| More time available for coding activities | 18 | 11 | 24 | 27 | 20 |  |  |
| Availability of continuous training | 11 | 14 | 26 | 33 | 16 |  |  |
| Availability of programs/software | 14 | 7 | 20 | 29 | 29 |  |  |
| Financial incentives according to quality | 18 | 6 | 13 | 18 | 45 |  |  |
| Frequent internal audits | 10 | 10 | 24 | 33 | 22 |  |  |
| Hospitals | 5 | 10 | 25 | 33 | 26 |  |  |
| Clinical specialties | 1 | 5 | 18 | 37 | 39 |  |  |
| Health professionals that make the record | 0 | 4 | 16 | 17 | 63 |  |  |
| Type of clinical record | 1 | 7 | 11 | 31 | 50 |  |  |
| Type of episode | 4 | 7 | 26 | 38 | 25 |  |  |
| Frequency of the event | 5 | 10 | 31 | 37 | 16 |  |  |
| COVID-19 pandemic | 14 | 15 | 31 | 25 | 16 |  |  |
| Principal diagnosis definition | 3 | 27 | 29 | 29 | 12 |  |  |
| Identification of secondary diagnosis and comorbidities | 4 | 27 | 37 | 28 | 4 |  |  |
| Definition of diagnostic priorities (in relation to the max. number of codes) | 8 | 22 | 39 | 27 | 4 |  |  |
| Coding of procedures | 4 | 25 | 35 | 26 | 10 |  |  |
| Quality of medical records | 0 | 0 | 3 | 7 | 90 |  |  |
| Interaction between the coders and the physicians in charge of the registers | 2 | 10 | 31 | 35 | 21 |  |  |
| Classification system used | 5 | 7 | 25 | 31 | 33 |  |  |
| Documents used to perform the coding (discharge note vs all documents) | 0 | 3 | 5 | 29 | 63 |  |  |
| Way of performing coding (paper vs electronic) | 5 | 4 | 18 | 31 | 42 |  |  |
| Coder training | 0 | 1 | 7 | 27 | 65 |  |  |
| Coder experience | 0 | 1 | 7 | 28 | 63 |  |  |
| Coder clinical knowledge | 0 | 3 | 7 | 31 | 59 |  |  |
| Utilization of standards in clinical records helps clinical coding | 1 | 7 | 35 | 34 | 24 |  |  |
| Creation of official acronym list helps clinical coding | 2 | 7 | 8 | 37 | 46 |  |  |
| Access to clinical records for clinical coding activity is not difficult | 14 | 27 | 14 | 26 | 19 |  |  |
| Available supporting material increases the precision of the coding process | 0 | 3 | 5 | 27 | 66 |  |  |
| Available software for coding increases the precision of the coding process | 3 | 2 | 10 | 27 | 59 |  |  |
| Coded data have enough quality for the purposes of hospital financing | 1 | 9 | 10 | 50 | 29 |  |  |
| Coded data have enough quality for the purposes of clinical research | 3 | 12 | 9 | 46 | 29 |  |  |
| ICD-10 will allow for more refined hospital reimbursement | 3 | 5 | 35 | 38 | 20 |  |  |
| ICD-10 will allow for more reliable clinical research | 3 | 7 | 24 | 41 | 26 |  |  |
| Utilization of standards in clinical records helps clinical coding | 1 | 7 | 35 | 34 | 24 |  |  |
| Creation of official acronym list helps clinical coding | 2 | 7 | 8 | 37 | 46 |  |  |
| Access to clinical records for clinical coding activity is not difficult | 14 | 27 | 14 | 26 | 19 |  |  |
| Available supporting material increases the precision of the coding process | 0 | 3 | 5 | 27 | 66 |  |  |
| Available software for coding increases the precision of the coding process | 3 | 2 | 10 | 27 | 59 |  |  |
| Coded data have enough quality for the purposes of hospital financing | 1 | 9 | 10 | 50 | 29 |  |  |
| Coded data have enough quality for the purposes of clinical research | 3 | 12 | 9 | 46 | 29 |  |  |
| ICD-10 complexity | 23 | 18 | 14 | 34 | 11 |  |  |
| Reduction of number of coders | 16 | 7 | 26 | 27 | 23 |  |  |
| No available training activities | 14 | 19 | 31 | 29 | 6 |  |  |
| Evolution of medical record quality since 2018 | 20 | 45 | 6 | 20 | 7 | 3 |  |
| Incomplete records | 0 | 9 | 67 | 23 | 1 |  |  |
| Differing information between records | 1 | 30 | 61 | 7 | 1 |  |  |
| No established diagnosis | 3 | 31 | 54 | 11 | 1 |  |  |
| No record of comorbidities | 0 | 16 | 53 | 30 | 1 |  |  |
| Unspecified acronym use | 0 | 8 | 41 | 44 | 7 |  |  |
| 'Copy-paste' use | 1 | 6 | 25 | 44 | 24 |  |  |
| Unreadable paper records | 40 | 24 | 23 | 12 | 2 |  |  |
| Unspecified diagnosis | 0 | 26 | 56 | 17 | 1 |  |  |
| Unavailable records at moment of coding | 18 | 42 | 33 | 5 | 1 |  |  |
| Health professionals are aware of the importance of clinical records for the coding process | 24 | 37 | 8 | 25 | 6 |  |  |
| When I ask the health professional responsible for the clinical record for clarifications, I get a quick and adequate reply | 17 | 29 | 17 | 30 | 7 |  |  |
| Health professionals are not motivated to produce adequately detailed information for the coding process | 5 | 7 | 9 | 48 | 31 |  |  |
| Health professionals have enough time to produce adequately detailed information for the coding process | 29 | 29 | 15 | 18 | 8 |  |  |
| The clinical terminology used by health professionals differs from the terminology used in the coding process | 5 | 17 | 12 | 49 | 17 |  |  |
| Health professionals with more experience produce better quality clinical records | 19 | 25 | 20 | 28 | 8 |  |  |
| Health professionals are more aware of the importance of recording the principal diagnosis than secondary diagnosis/comorbidities | 7 | 12 | 18 | 37 | 25 |  |  |
| Incomplete records | 0 | 1 | 1 | 18 | 80 |  |  |
| Differing information between records | 0 | 1 | 1 | 22 | 75 |  |  |
| No established diagnosis | 1 | 1 | 3 | 22 | 74 |  |  |
| No record of comorbidities | 0 | 1 | 2 | 30 | 67 |  |  |
| Unspecified acronym use | 0 | 2 | 6 | 43 | 49 |  |  |
| 'Copy-paste' use | 3 | 3 | 12 | 37 | 46 |  |  |
| Unreadable paper records | 15 | 3 | 16 | 11 | 55 |  |  |
| Unspecified diagnosis | 1 | 3 | 10 | 29 | 58 |  |  |
| Unavailable records at moment of coding | 4 | 5 | 15 | 24 | 52 |  |  |
| How often do you receive feedback on the quality of your data? | 31 | 6 | 23 | 12 | 8 | 17 | 3 |
| How often do you meet with an auditor to assess the quality of your data? | 41 | 10 | 20 | 7 | 11 | 8 | 3 |
| How often do you consider it relevant to have continuing education? | 1 | 33 | 5 | 17 | 13 | 26 | 5 |
| It makes the coding process faster | 6 | 7 | 5 | 20 | 62 |  |  |
| It makes the coding process simpler | 5 | 3 | 5 | 14 | 73 |  |  |
| It increases the quality of the coded data | 6 | 1 | 5 | 14 | 74 |  |  |

2. **Inferential statistical analysis – Stepwise logistic regressions**

Table S3. Areas of clinical coding process analyzed in the logistic regression models

| Dimension | Outputs |
| --- | --- |
| Factors impacting quality of health records | Hospitals |
|  | Clinical specialties |
|  | Health professional |
|  | Type of clinical record |
|  | Type of episode |
|  | Frequency of event |
| Frequency of problems in health records | Incomplete records |
|  | Different info between records |
|  | No established diagnosis |
|  | No record of comorbities |
|  | Unspecified acronym use |
|  | Copy-paste' use |
|  | Unreadable paper records |
|  | Unspecified diagnosis |
|  | Unavailable records |
| Negative impact on coding quality | Incomplete records |
|  | Different info between records |
|  | No established diagnosis |
|  | No record of comorbities |
|  | Unspecified acronym use |
|  | Copy-paste' use |
|  | Unreadable paper records |
|  | Unspecified diagnosis |
|  | Unavailable records |
| Factors impacting clinical coding | Sentence A: Utilization of standards in clinical records helps clinical coding. |
|  | Sentence B: Creation of an official acronym list helps clinical coding. |
|  | Sentence C: Access to clinical records for clinical coding activity is not difficult. |
|  | Sentence D: Available support material increases the precision of the coding process. |
|  | Sentence E: Available software for coding increases the precision of the coding process. |
|  | Sentence F: Coded data have enough quality for the purpose of hospital financing. |
|  | Sentence G: Coded data have enough quality for the purpose of clinical research. |
|  | Sentence H: ICD-10 will allow for more reliable clinical research. |
|  | Sentence I: ICD-10 will allow for more refined hospital reimbursement. |
| Difficulties on clinical coding | Principal diagnosis definition |
|  | Identification of second diagnosis |
|  | Coding of procedures |
|  | Definition of diagnostic priorities |
| Contribution of professionals | Sentence A: Health professionals are aware of the importance of clinical records for the coding process. |
|  | Sentence B: When I ask the health professional responsible for the clinical record for clarifications, I get a quick and adequate reply. |
|  | Sentence C: Health professionals are not motivated to produce adequately detailed information for the coding process. |
|  | Sentence D: Health professionals have enough time to produce adequately detailed information for the coding process. |
|  | Sentence E: The clinical terminology used by health professionals differs from the terminology used in the coding process. |
|  | Sentence F: Health professionals with more clinical experience produce better quality clinical records. |
|  | Sentence G: Health professionals are more aware of the importance of recoding the principal diagnosis than recording secondary diagnoses/comorbidities. |
| Electronical coding | It increases quality of coded data |
|  | It makes the coding process simpler |
|  | It makes the coding process faster |

(continues)

(conclusion)

| Dimension | Outputs |
| --- | --- |
| Causes for delay in coding | Reduction of number of coders |
|  | ICD-10 complexity |
|  | No available training activities |
| Priority for increasing data quality | Financial incentives |
|  | Availability of programs/software |
|  | Frequent internal audits |
|  | More time for coding activities |
| Factors impacting coding quality | Quality of medical records |
|  | Coder training |
|  | Documents used to perform the coding |
|  | Coder experience |
|  | Coder clinical knowledge |
|  | Way of performing coding |
|  | Classification system used |
|  | Interaction between coders and physicians in charge of registers |

Table S4. Odds ratios (OR) of stepwise logistic regressions on factors impacting quality of health records

| Variables | Factors impacting quality of health records | | | | | |
| --- | --- | --- | --- | --- | --- | --- |
|  | Hospitals | Clinical specialties | Health professional | Type of clinical record | Type of episode | Frequency of event |
| Intercept | 0.00 | 0.00 | 0.00 | 0.00 | 0.00 | 207.37 |
| Sex | 1.80 |  |  |  |  |  |
| Age | 0.50 |  | **4.52** | 2.03 |  |  |
| Experience |  |  |  |  |  |  |
| Weekly working hours | 1.91 |  |  |  |  |  |
| Region of work - Center | **0.24** | 0.50 | 2.11 | 2.58 | 0.51 |  |
| Region of work - LVT | 1.19 | 1.21 | 3.43 | 2.21 | 1.27 |  |
| Region of work - Other | **0.23** | **0.19** | 0.42 | 0.33 | 0.35 |  |
| Clinical field - Surgical | 0.94 | 1.14 |  |  |  | 0.66 |
| Clinical field - Medical-Surgical | **4.74** | **0.28** |  |  |  | 0.39 |
| Clinical field - Other | 6.10 | 0.25 |  |  |  | 6.07 |
| Field of expertise - Surgical | **5.60** |  |  |  |  |  |
| Field of expertise - Medical-Surgical | 5.84E+07 |  |  |  |  |  |
| Field of expertise - Other | 5.17 |  |  |  |  |  |
| Same department |  |  |  | **0.23** |  | **0.25** |
| Same hospital |  | **4.44** |  |  |  | **0.35** |
| Different hospital |  | **5.47** |  |  |  |  |
| Coding same field of expertise | **8.46** |  |  | 2.81 |  |  |
| Type of register - EHR | 2.42E+07 | 1.38E+15 | 1.69E+08 | 3.26E+09 | 1.77E+07 | 1.89E+07 |
| Type of register - HR | 0.39 | 0.34 |  |  |  |  |
| Type of register - Other |  |  | 0.00 |  |  |  |
| Type of register - Both |  |  |  |  |  |  |
| Documents used - Discharge note |  |  |  |  | 7.12E+07 | 0.00 |
| Documents used - Clinical diary |  |  |  |  | 0.21 |  |
| Documents used - Anesthetic report | 0.55 |  |  |  |  |  |
| Documents used - Surgical report |  |  |  | 0.10 |  | 0.32 |
| Documents used - Complementary Diagnostic Tests Report |  | 2.01 |  |  |  |  |
| Documents used - Nursing record |  |  |  |  |  |  |
| Documents used - Clinical summary |  |  |  |  | **0.35** |  |
| Coding registry - Paper | **7.93** | 2.64 |  | 2.50 | **9.18** | 2.87 |
| Coding registry - Both | 2.04 | **12.90** |  | 1.88E+07 | 3.63 | 0.39 |
| Software preference - SIMH |  | 0.12 | 0.36 |  | 3.04 |  |
| Software preference - Other |  | 0.15 |  |  |  |  |
| Auditing - External only |  | 9.08E+06 |  |  |  | 0.13 |
| Auditing - Both |  | 0.28 |  |  |  | 1.09 |
| Auditing - do not know/neither |  | 0.35 |  |  |  | 0.50 |
| Delay - Between 2 and 6 months |  |  |  |  |  |  |
| Delay - Unknown |  |  |  |  |  |  |

Note: Values highlighted in green denote a positive association (OR>1) and those highlighted in orange denote a negative association (OR<1). Values in bold indicate OR for which p<0.05.

Table S5. Odds ratios (OR) of stepwise logistic regressions on frequency of problems in health records

| Variables | Frequency of problems in health records | | | | | | | | |
| --- | --- | --- | --- | --- | --- | --- | --- | --- | --- |
|  | Incom-plete records | Different info between records | No established diagnosis | No record of comorbities | Unspecified acronym use | 'Copy-paste' use | Unreadable paper records | Unspecified diagnosis | Unavailable records |
| Intercept | 0.00 | 0.00 | 0.00 | 0.00 | 0.37 | 0.00 | 0.00 | 0.00 | 0.00 |
| Sex |  |  |  |  |  |  |  |  |  |
| Age |  |  |  |  |  |  |  |  |  |
| Experience |  |  |  |  |  |  |  |  |  |
| Weekly working hours |  |  |  |  |  |  |  |  |  |
| Region of work - Center |  |  |  |  |  |  |  |  |  |
| Region of work - LVT |  |  |  |  |  |  |  |  |  |
| Region of work - Other |  |  |  |  |  |  |  |  |  |
| Clinical field - Surgical |  |  |  |  |  |  |  |  |  |
| Clinical field - Medical-Surgical |  |  |  |  |  |  |  |  |  |
| Clinical field - Other |  |  |  |  |  |  |  |  |  |
| Field of expertise - Surgical |  |  |  |  |  |  |  |  |  |
| Field of expertise - Medical-Surgical |  |  |  |  |  |  |  |  |  |
| Field of expertise - Other |  |  |  |  |  |  |  |  |  |
| Same department |  |  |  |  |  |  |  |  |  |
| Same hospital |  |  |  |  |  |  |  |  |  |
| Different hospital |  |  |  |  |  |  |  |  |  |
| Coding same field of expertise |  |  |  |  |  |  |  |  |  |
| Type of register - EHR |  |  |  |  |  |  |  |  |  |
| Type of register - HR |  |  |  |  |  |  |  |  |  |
| Type of register - Other |  |  |  |  | 1.61E+07 |  |  |  |  |
| Type of register - Both |  |  |  |  |  |  |  |  |  |
| Documents used - Discharge note |  |  |  |  |  |  |  |  |  |
| Documents used - Clinical diary |  |  |  |  | 3.20 |  |  |  |  |
| Documents used - Anesthetic report |  |  |  |  |  |  |  |  |  |
| Documents used - Surgical report |  |  |  |  |  |  |  |  |  |
| Documents used - Complementary Diagnostic Tests Report |  |  |  |  |  |  |  |  |  |
| Documents used - Nursing record |  |  |  |  |  |  |  |  |  |
| Documents used - Clinical summary |  |  |  |  | 0.36 |  |  |  |  |
| Coding registry - Paper |  |  |  |  | **0.31** |  |  |  |  |
| Coding registry - Both |  |  |  |  | 0.75 |  |  |  |  |
| Software preference - SIMH |  |  |  |  |  |  |  |  |  |
| Software preference - Other |  |  |  |  |  |  |  |  |  |
| Auditing - External only |  |  |  |  |  |  |  |  |  |
| Auditing - Both |  |  |  |  |  |  |  |  |  |
| Auditing - do not know/neither |  |  |  |  |  |  |  |  |  |
| Delay - Between 2 and 6 months |  |  |  |  |  |  |  |  |  |
| Delay - Unknown |  |  |  |  |  |  |  |  |  |

Note: Values highlighted in green denote a positive association (OR>1) and those highlighted in orange denote a negative association (OR<1). Values in bold indicate OR for which p<0.05.

Table S6. Odds ratios (OR) of stepwise logistic regressions on negative impact of problems in health records

| Variables | Negative impact on coding quality | | | | | | | | |
| --- | --- | --- | --- | --- | --- | --- | --- | --- | --- |
|  | Incom-plete records | Different info between records | No established diagnosis | No record of comor-  bities | Unspecified acronym  use | 'Copy-paste' use | Unreada-ble paper records | Unspecified diagnosis | Unavai-  lable records |
| Intercept |  | 7.55E+212 |  | 1.60E+142 | 4.46E+237 | **10.51** | **7.04** | **26.38** | **66.63** |
| Sex |  |  | 0.00 | 0.00 | 0.00 |  |  | 0.40 | 0.50 |
| Age | 0.00 | 0.00 | 2.08E+15 |  |  | **0.43** | 0.46 | **0.19** | **0.27** |
| Experience |  |  |  |  |  |  | 0.50 |  |  |
| Weekly working hours | 0.00 | 0.00 | 0.00 | 0.00 |  |  |  |  |  |
| Region of work - Center |  |  |  |  | 0.00 |  |  |  | 0.20 |
| Region of work - LVT |  |  |  |  |  |  |  |  |  |
| Region of work - Other |  |  |  |  | 0.00 |  |  |  | 0.26 |
| Clinical field - Surgical |  |  |  |  |  |  |  |  | 2.17 |
| Clinical field - Medical-Surgical |  |  |  |  |  |  |  |  | 1.75 |
| Clinical field - Other |  |  |  |  |  |  |  |  | **0.08** |
| Field of expertise - Surgical |  |  |  |  |  |  |  |  |  |
| Field of expertise - Medical-Surgical |  |  |  |  | 0.00 |  |  |  |  |
| Field of expertise - Other |  |  |  |  | 1.72E+70 |  |  |  |  |
| Same department |  | 2.16E+68 |  | 0.00 | 0.00 | 0.45 |  |  | **0.15** |
| Same hospital |  |  | 0.00 | 0.00 |  |  | 0.50 |  | **0.18** |
| Different hospital |  |  |  | 4.95E+67 | 0.00 | 0.40 |  |  | 0.22 |
| Coding same field of expertise | 0.00 | 0.00 | 0.00 | 1.35E+33 |  |  |  |  | 0.35 |
| Type of register - EHR |  |  |  |  |  |  |  |  |  |
| Type of register - HR |  |  | 6.39E+202 |  |  |  | 2.34 |  | **3.78** |
| Type of register - Other |  |  |  |  |  |  |  |  |  |
| Type of register - Both |  |  |  |  |  |  |  |  |  |
| Documents used - Discharge note |  |  |  |  |  |  |  |  |  |
| Documents used - Clinical diary |  |  |  |  |  |  |  |  |  |
| Documents used - Anesthetic report | 3.73E+18 | 3.09E+33 | 2.98E+279 | 1.03E+50 | 0.00 |  |  |  | 0.40 |
| Documents used - Surgical report | 0.00 |  | 0.00 |  |  |  |  |  |  |
| Documents used - Complementary Diagnostic Tests Report |  | 0.00 |  |  | 0.00 |  |  |  |  |
| Documents used - Nursing record |  | 4.90E+51 | 1.45E+29 |  | 2.61E+68 |  |  |  |  |
| Documents used - Clinical summary | 0.00 | 0.00 | 0.00 | 0.00 | 0.00 |  | **0.22** |  | **0.18** |
| Coding registry - Paper |  |  | 0.00 |  | 0.00 |  |  |  |  |
| Coding registry - Both |  |  | 0.00 |  | 5.05E+84 |  |  |  |  |
| Software preference - SIMH |  |  |  | 5.90E+37 |  |  |  |  |  |
| Software preference - Other |  |  | 1.33E+108 |  |  |  | 2.06 |  | 3.66 |
| Auditing - External only |  |  |  |  |  |  |  |  | 4.95E+07 |
| Auditing - Both |  |  |  |  |  |  |  |  | 0.63 |
| Auditing - do not know/neither |  |  |  |  |  |  |  |  | 0.65 |
| Delay - Between 2 and 6 months |  |  |  |  |  |  | 3.56 |  | 2.88 |
| Delay - Unknown |  |  |  |  |  |  | 0.62 |  | **4.17** |

Note: Values highlighted in green denote a positive association (OR>1) and those highlighted in orange denote a negative association (OR<1). Values in bold indicate OR for which p<0.05.

Table S7. Odds ratios (OR) of stepwise logistic regressions on factors impacting clinical coding

| Variables | Factors impacting clinical coding | | | | | | | | |
| --- | --- | --- | --- | --- | --- | --- | --- | --- | --- |
|  | Sentence A | Sentence B | Sentence C | Sentence  D | Sentence E | Sentence F | Sentence G | Sentence H | Sentence I |
| Intercept | 0.00 | 0.00 | 0.00 |  | 0.00 | **47.29** | **157.42** | 0.00 | 0.00 |
| Sex | 0.52 | **0.37** |  | 8.91E+172 | **0.20** |  | **0.30** | 2.16 |  |
| Age |  | 2.20 |  |  |  |  |  | **0.34** |  |
| Experience |  |  |  | 0.00 |  |  |  | 0.49 |  |
| Weekly working hours | 1.81 |  | 1.61 | 1.39E+158 |  |  |  |  |  |
| Region of work - Center |  |  |  |  |  | 1.73 | 1.12 | 2.16 |  |
| Region of work - LVT |  |  |  |  |  | **0.31** | **0.32** | 0.48 |  |
| Region of work - Other |  |  |  |  |  | **0.22** | **0.20** | 0.61 |  |
| Clinical field - Surgical |  |  |  |  | **6.39** |  |  |  |  |
| Clinical field - Medical-Surgical |  |  |  |  | 1.20 |  |  |  |  |
| Clinical field - Other |  |  |  |  | 0.60 |  |  |  |  |
| Field of expertise - Surgical |  |  |  | 0.00 |  |  |  |  |  |
| Field of expertise - Medical-Surgical |  |  |  | 0.00 |  |  |  |  |  |
| Field of expertise - Other |  |  |  | 0.00 |  |  |  |  |  |
| Same department |  | 4.08 |  |  |  |  | **0.33** |  |  |
| Same hospital |  |  |  | 5.02E+157 |  |  |  |  |  |
| Different hospital |  |  |  | 1.71E+272 |  | 2.58 |  |  |  |
| Coding same field of expertise |  |  |  | 0.00 |  |  |  |  |  |
| Type of register - EHR | 4.54E+06 | 1.82E+08 | 4.39E+06 |  |  |  |  |  |  |
| Type of register - HR | **0.46** |  |  | 9.97E+194 |  |  | **3.62** |  |  |
| Type of register - Other |  |  |  |  |  |  |  | 3.62E+07 |  |
| Type of register - Both |  |  |  |  |  |  |  |  |  |
| Documents used - Discharge note |  | 1.05E+09 |  |  |  |  |  | 3.15E+08 | 4.19E+07 |
| Documents used - Clinical diary |  |  |  | 3.93E+302 |  | 0.24 |  | **0.06** | 0.27 |
| Documents used - Anesthetic report |  |  | 1.65 | 7.77E+243 | **11.55** |  | 2.10 |  |  |
| Documents used - Surgical report |  |  |  | 0.00 |  |  | **0.08** |  |  |
| Documents used - Complementary Diagnostic Tests Report |  |  | 1.65 | 0.00 |  |  |  | **2.23** |  |
| Documents used - Nursing record |  |  |  | 1.75E+100 |  | 0.46 | **0.32** |  |  |
| Documents used - Clinical summary |  |  |  | 0.00 |  |  |  | 0.41 |  |
| Coding registry - Paper |  | 0.56 |  |  | **0.01** |  |  | 2.30 |  |
| Coding registry - Both |  | 1.84E+07 |  |  | 1.08 |  |  | **8.00** |  |
| Software preference - SIMH |  |  |  | 7.46E+155 | 3.42E+07 |  |  |  |  |
| Software preference - Other |  |  |  |  | 4.72E+07 | **0.22** | **0.24** | **0.22** | **0.45** |
| Auditing - External only |  |  |  | 1.82E+82 |  |  |  |  |  |
| Auditing - Both |  |  |  |  |  |  |  |  |  |
| Auditing - do not know/neither |  |  |  | 5.12E+150 |  |  |  |  |  |
| Delay - Between 2 and 6 months |  | 0.33 |  |  |  |  |  | 1.15 | 0.60 |
| Delay - Unknown |  | 1.14 |  |  |  |  |  | 0.44 | **0.31** |

Note: Values highlighted in green denote a positive association (OR>1) and those highlighted in orange denote a negative association (OR<1). Values in bold indicate OR for which p<0.05.

Table S8. Odds ratios (OR) of stepwise logistic regressions on difficulties in clinical coding

| Variables | Difficulties in clinical coding | | | |
| --- | --- | --- | --- | --- |
|  | Principal diagnosis definition | Identification of second diagnosis | Coding of procedures | Definition of diagnostic priorities |
| Intercept | 0.54 | 3.55E+07 | 1.51 | 1.81 |
| Sex |  |  | 0.54 |  |
| Age | **0.41** | **0.24** | **2.83** | 0.58 |
| Experience |  |  |  |  |
| Weekly working hours |  |  |  |  |
| Region of work - Center |  |  | **8.20** |  |
| Region of work - LVT |  |  | 1.31 |  |
| Region of work - Other |  |  | 2.43 |  |
| Clinical field - Surgical |  |  |  |  |
| Clinical field - Medical-Surgical |  |  |  |  |
| Clinical field - Other |  |  |  |  |
| Field of expertise - Surgical | 0.52 |  |  |  |
| Field of expertise - Medical-Surgical | 2.11E+07 |  |  |  |
| Field of expertise - Other | 3.34 |  |  |  |
| Same department | **0.33** |  | **0.14** | 0.40 |
| Same hospital |  |  | 0.36 | 0.29 |
| Different hospital |  |  | **0.25** | 0.29 |
| Coding same field of expertise | 4.90 |  |  |  |
| Type of register - EHR |  | 0.00 |  |  |
| Type of register - HR |  | 2.21 |  | 2.00 |
| Type of register - Other | 4.03E+07 |  | 3.18E+07 |  |
| Type of register - Both |  |  |  |  |
| Documents used - Discharge note |  |  |  |  |
| Documents used - Clinical diary |  |  |  |  |
| Documents used - Anesthetic report |  | 2.23 |  |  |
| Documents used - Surgical report |  | 0.29 |  |  |
| Documents used - Complementary Diagnostic Tests Report |  |  |  |  |
| Documents used - Nursing record |  |  |  |  |
| Documents used - Clinical summary |  |  | **0.26** |  |
| Coding registry - Paper |  |  | **0.05** |  |
| Coding registry - Both |  |  | 0.35 |  |
| Software preference - SIMH |  |  | **0.18** |  |
| Software preference - Other | 0.50 | 0.39 |  |  |
| Auditing - External only |  | 0.00 | 3.18 |  |
| Auditing - Both |  | 1.51 | **5.56** |  |
| Auditing - do not know/neither |  | 0.86 | 2.93 |  |
| Delay - Between 2 and 6 months | **0.27** |  | 0.66 |  |
| Delay - Unknown | 1.00 |  | 2.34 |  |

Note: Values highlighted in green denote a positive association (OR>1) and those highlighted in orange denote a negative association (OR<1). Values in bold indicate OR for which p<0.05.

Table S9. Odds ratios (OR) of stepwise logistic regressions on contribution of health professionals

| Variables | Contribution of health professionals | | | | | | |
| --- | --- | --- | --- | --- | --- | --- | --- |
|  | Sentence A | Sentence B | Sentence C | Sentence D | Sentence E | Sentence F | Sentence G |
| Intercept | 0.00 | 0.05 | 7.77E+07 | 3.60E+13 | 1.59E+07 | 1.28E+07 | **5.33** |
| Sex | **0.33** |  |  |  |  |  | **2.25** |
| Age |  |  |  | **0.25** |  | **0.17** | **0.23** |
| Experience | 1.75 |  |  | **0.28** |  | 0.49 | 0.43 |
| Weekly working hours |  |  |  |  |  | 1.89 |  |
| Region of work - Center |  |  |  |  |  |  |  |
| Region of work - LVT |  |  |  |  |  |  |  |
| Region of work - Other |  |  |  |  |  |  |  |
| Clinical field - Surgical |  | **0.13** | 5.40 |  |  |  |  |
| Clinical field - Medical-Surgical |  | 0.39 | 7.50 |  |  |  |  |
| Clinical field - Other |  | 1.93 | 0.81 |  |  |  |  |
| Field of expertise - Surgical |  | **8.06** | 0.19 |  |  |  |  |
| Field of expertise - Medical-Surgical |  | 5.65E+05 | 0.00 |  |  |  |  |
| Field of expertise - Other |  | 2.68 | 0.00 |  |  |  |  |
| Same department |  | **0.08** |  | **4.33** |  |  |  |
| Same hospital |  | 0.12 |  |  |  |  |  |
| Different hospital |  | **0.04** |  |  | **0.32** |  | 0.46 |
| Coding same field of expertise |  |  | 0.00 |  | **0.42** | 0.54 | **0.45** |
| Type of register - EHR | 7.26E+06 |  |  |  | 0.00 | 0.00 |  |
| Type of register - HR | **2.44** |  |  |  | **0.29** |  |  |
| Type of register - Other |  | 0.00 |  |  | 0.00 |  |  |
| Type of register - Both |  |  |  |  |  |  |  |
| Documents used - Discharge note |  |  |  | 0.00 |  |  |  |
| Documents used - Clinical diary |  | 4.86 |  |  |  |  |  |
| Documents used - Anesthetic report |  |  |  | **0.34** | 0.48 |  | 1.76 |
| Documents used - Surgical report |  | 0.25 | **3.90** | **7.31** |  |  |  |
| Documents used - Complementary Diagnostic Tests Report |  | **2.55** |  |  |  |  |  |
| Documents used - Nursing record | **0.44** |  |  | **2.95** | **2.84** |  |  |
| Documents used - Clinical summary | **2.67** | **3.45** | **0.30** | 0.41 |  |  | **0.30** |
| Coding registry - Paper |  |  |  |  |  |  |  |
| Coding registry - Both |  |  |  |  |  |  |  |
| Software preference - SIMH |  | **45.23** |  |  |  | 2.35 |  |
| Software preference - Other |  | **36.41** |  |  | **2.79** |  |  |
| Auditing - External only |  |  |  | 0.00 |  |  |  |
| Auditing - Both |  |  |  | **3.58** |  |  |  |
| Auditing - do not know/neither |  |  |  | 1.40 |  |  |  |
| Delay - Between 2 and 6 months |  |  |  | 3.71 |  |  |  |
| Delay - Unknown |  |  |  | 2.62 |  |  |  |

Note: Values highlighted in green denote a positive association (OR>1) and those highlighted in orange denote a negative association (OR<1). Values in bold indicate OR for which p<0.05.

Table S10. Odds ratios (OR) of stepwise logistic regressions on electronic coding

| Variables | Electronic coding | | |
| --- | --- | --- | --- |
|  | It increases quality of coded data | It makes the coding process simpler | It makes the coding process faster |
| Intercept | **974.25** | 4.03E+08 | 4.84E+07 |
| Sex |  | 0.45 | 0.38 |
| Age |  |  |  |
| Experience |  |  |  |
| Weekly working hours |  | 2.47 |  |
| Region of work - Center |  |  |  |
| Region of work - LVT |  |  |  |
| Region of work - Other |  |  |  |
| Clinical field - Surgical |  |  |  |
| Clinical field - Medical-Surgical |  |  |  |
| Clinical field - Other |  |  |  |
| Field of expertise - Surgical |  |  |  |
| Field of expertise - Medical-Surgical |  |  |  |
| Field of expertise - Other |  |  |  |
| Same department |  |  | 4.24 |
| Same hospital |  |  | 2.42 |
| Different hospital |  | 0.38 |  |
| Coding same field of expertise |  |  |  |
| Type of register - EHR |  |  |  |
| Type of register - HR |  |  |  |
| Type of register - Other | 1.19E+07 |  |  |
| Type of register - Both |  |  |  |
| Documents used - Discharge note |  |  |  |
| Documents used - Clinical diary | 0.22 | 0.00 | 0.00 |
| Documents used - Anesthetic report |  |  |  |
| Documents used - Surgical report |  |  |  |
| Documents used - Complementary Diagnostic Tests Report |  |  |  |
| Documents used - Nursing record |  |  |  |
| Documents used - Clinical summary | **0.30** |  |  |
| Coding registry - Paper | **0.06** | **0.10** |  |
| Coding registry - Both | 3.04 | 4.46E-01 |  |
| Software preference - SIMH | **0.03** |  |  |
| Software preference - Other | **0.11** |  | **0.25** |
| Auditing - External only |  |  |  |
| Auditing - Both |  |  |  |
| Auditing - do not know/neither |  |  |  |
| Delay - Between 2 and 6 months |  |  |  |
| Delay - Unknown |  |  |  |

Note: Values highlighted in green denote a positive association (OR>1) and those highlighted in orange denote a negative association (OR<1). Values in bold indicate OR for which p<0.05.

Table S11. Odds ratios (OR) of stepwise logistic regressions on causes of delay in coding

| Variables | Causes of delay in coding | | |
| --- | --- | --- | --- |
|  | Reduction of number of coders | ICD-10 complexity | No available training activities |
| Intercept | **0.06** | 0.00 | 0.40 |
| Sex | **0.39** |  |  |
| Age |  |  |  |
| Experience | 1.80 | 0.54 |  |
| Weekly working hours |  |  | **0.50** |
| Region of work - Center |  | 2.16 |  |
| Region of work - LVT |  | **3.91** |  |
| Region of work - Other |  | 2.40 |  |
| Clinical field - Surgical |  |  |  |
| Clinical field - Medical-Surgical |  |  |  |
| Clinical field - Other |  |  |  |
| Field of expertise - Surgical |  |  |  |
| Field of expertise - Medical-Surgical |  |  |  |
| Field of expertise - Other |  |  |  |
| Same department |  | 0.54 |  |
| Same hospital | 2.06 |  |  |
| Different hospital |  | **0.28** |  |
| Coding same field of expertise |  |  |  |
| Type of register - EHR |  |  |  |
| Type of register - HR | 0.49 | **0.37** |  |
| Type of register - Other | 0.00 |  | 5.42E+06 |
| Type of register - Both |  |  |  |
| Documents used - Discharge note |  | 9.45E+06 |  |
| Documents used - Clinical diary | **5.77** |  |  |
| Documents used - Anesthetic report |  | **3.24** |  |
| Documents used - Surgical report |  |  | 2.56 |
| Documents used - Complementary Diagnostic Tests Report |  |  |  |
| Documents used - Nursing record |  |  |  |
| Documents used - Clinical summary |  |  |  |
| Coding registry - Paper |  |  | 0.38 |
| Coding registry - Both |  |  | **0.12** |
| Software preference - SIMH |  | 6.59 |  |
| Software preference - Other | **2.36** | 5.93 |  |
| Auditing - External only | 0.68 |  |  |
| Auditing - Both | **3.11** |  |  |
| Auditing - do not know/neither | 1.34 |  |  |
| Delay - Between 2 and 6 months |  |  |  |
| Delay – Unknown |  |  |  |

Note: Values highlighted in green denote a positive association (OR>1) and those highlighted in orange denote a negative association (OR<1). Values in bold indicate OR for which p<0.05.

Table S12. Odds ratios (OR) of stepwise logistic regressions on priorities regarding coding process

| Variables | Priorities regarding coding process | | | | |
| --- | --- | --- | --- | --- | --- |
|  | Financial incentives | Availability of programs/software | Frequent internal audits | Availability of continuous training | More time for coding activities |
| Intercept | **0.05** | 1.99E+05 | **0.10** | 0.23 | 6.37E+06 |
| Sex | **2.43** |  |  | **2.25** |  |
| Age |  |  |  |  | 0.57 |
| Experience | 0.56 |  |  | 1.97 |  |
| Weekly working hours |  | **2.72** |  | 0.57 |  |
| Region of work - Center |  |  |  |  |  |
| Region of work - LVT |  |  |  |  |  |
| Region of work - Other |  |  |  |  |  |
| Clinical field - Surgical | **2.50** |  | 2.42 |  |  |
| Clinical field - Medical-Surgical | 0.53 |  | 1.73 |  |  |
| Clinical field - Other | 2.33 |  | 8.92 |  |  |
| Field of expertise - Surgical |  |  | 1.71 |  |  |
| Field of expertise - Medical-Surgical |  |  | 0.00 |  |  |
| Field of expertise - Other |  |  | 0.54 |  |  |
| Same department | **6.60** | **3.81** |  | 2.95 |  |
| Same hospital |  |  |  | 2.37 |  |
| Different hospital |  |  |  | **3.81** |  |
| Coding same field of expertise | 0.45 | 1.94 |  |  |  |
| Type of register - EHR |  |  |  |  |  |
| Type of register - HR |  | 0.43 |  |  |  |
| Type of register - Other |  |  | 0.00 | 0.00 | 0.00 |
| Type of register - Both |  |  |  |  |  |
| Documents used - Discharge note |  | 0.00 |  |  | 0.00 |
| Documents used - Clinical diary | **5.25** |  | **4.96** |  |  |
| Documents used - Anesthetic report |  |  |  |  |  |
| Documents used - Surgical report |  | **5.07** | 3.15 |  | 2.51 |
| Documents used - Complementary Diagnostic Tests Report |  |  | 0.50 |  |  |
| Documents used - Nursing record |  |  | 1.90 |  |  |
| Documents used - Clinical summary | 2.40 |  | 2.28 |  |  |
| Coding registry - Paper | 3.71 | **3.14** |  | 4.61 | **3.58** |
| Coding registry - Both | 2.23 | 2.10 |  | **7.22** | 0.76 |
| Software preference - SIMH | 2.35 |  |  | 2.65 |  |
| Software preference - Other |  |  |  |  |  |
| Auditing - External only |  | 0.55 |  |  | 0.36 |
| Auditing - Both |  | **3.16** |  |  | **2.79** |
| Auditing - do not know/neither |  | 1.81 |  |  | 0.97 |
| Delay - Between 2 and 6 months |  |  |  | 1.12 |  |
| Delay - Unknown |  |  |  | **0.39** |  |

Note: Values highlighted in green denote a positive association (OR>1) and those highlighted in orange denote a negative association (OR<1). Values in bold indicate OR for which p<0.05.

Table S13. Odds ratios (OR) of stepwise logistic regressions on factors impacting coding quality

| Variables | Factors impacting coding quality | | | | | | | |
| --- | --- | --- | --- | --- | --- | --- | --- | --- |
|  | Quality of medical records | Coder training | Documents used to perform the coding | Coder experience | Coder clinical knowledge | Way of performing coding | Classification system used | Interaction between coders and physicians in charge of registers |
| Intercept | 0.00 | 0.00 | 7.77 | 0.00 | **4.70** | 3.26E+05 | 0.00 | 0.00 |
| Sex |  |  |  |  |  |  | 5.34E+87 |  |
| Age | 0.00 | **0.39** |  | 0.00 | 0.55 |  |  |  |
| Experience | 0.00 |  |  | 0.00 |  |  |  |  |
| Weekly working hours | 1.54E+18 |  |  |  |  |  |  |  |
| Region of work - Center | 2.42E+52 |  |  |  |  |  |  | 7.68E+07 |
| Region of work - LVT | 3.38E+17 |  |  |  |  |  |  | 2.12 |
| Region of work - Other | 3.04E+54 |  |  |  |  |  |  | 1.09E+08 |
| Clinical field - Surgical | 3.80E+35 |  |  | 0.00 |  |  | 0.00 |  |
| Clinical field - Medical-Surgical | 3.91E+38 |  |  | 0.00 |  |  | 0.00 |  |
| Clinical field - Other | 280.36 |  |  |  |  |  |  |  |
| Field of expertise - Surgical | 5.84E+53 |  |  |  |  |  |  |  |
| Field of expertise - Medical-Surgical | 1.56E+57 |  |  | 0.00 |  |  | 0.00 |  |
| Field of expertise - Other | 6.26E+72 |  |  |  |  |  | 6.37E+262 |  |
| Same department |  |  |  | 0.00 |  |  | 0.00 |  |
| Same hospital |  |  |  | 0.00 |  |  | 0.00 |  |
| Different hospital |  |  |  | 0.00 | 2.43 |  | 0.00 |  |
| Coding same field of expertise | 8.06E+90 |  |  | 0.00 |  |  |  |  |
| Type of register - EHR | 6.26E+98 | 4.10E+07 |  |  |  | **116.39** |  | 2.18E+08 |
| Type of register - HR | 1.12E+19 |  | 0.46 | 0.00 |  |  |  | **0.16** |
| Type of register - Other |  |  |  |  |  |  |  |  |
| Type of register - Both |  |  |  |  |  |  |  |  |
| Documents used - Discharge note |  |  |  |  |  |  |  | 3.19E+09 |
| Documents used - Clinical diary |  |  | 0.14 |  |  |  |  |  |
| Documents used - Anesthetic report |  | 0.54 |  |  |  |  |  |  |
| Documents used - Surgical report |  |  |  | 0.00 |  | 0.00 | 0.00 |  |
| Documents used - Complementary Diagnostic Tests Report |  |  |  |  |  | 4.68 |  |  |
| Documents used - Nursing record |  |  |  | 0.00 |  |  | 6.22E+261 | 3.29 |
| Documents used - Clinical summary |  | 2.84 |  | 0.00 |  |  | 0.00 |  |
| Coding registry - Paper |  | **4.70** |  |  | **0.28** |  | 0.00 |  |
| Coding registry - Both |  | 0.35 |  |  | **0.21** |  |  |  |
| Software preference - SIMH |  |  |  |  |  |  |  |  |
| Software preference - Other |  |  |  |  |  | 6.95 |  | 3.73 |
| Auditing - External only |  | 0.51 | 0.72 |  |  | 8.43 | 0.00 |  |
| Auditing - Both |  | **2.90** | 1.87 | 1.24E+293 |  | **20.78** |  |  |
| Auditing - do not know/neither |  | **3.62** | **3.05** | 0.00 |  | 4.46 | 0.00 |  |
| Delay - Between 2 and 6 months |  | 0.37 |  |  |  |  | 0.00 |  |
| Delay - Unknown |  | 0.43 |  |  |  |  | 1.08E+262 |  |

Note: Values highlighted in green denote a positive association (OR>1) and those highlighted in orange denote a negative association (OR<1). Values in bold indicate OR for which p<0.05.

3. **STROBE checklist**

|  | Item Description | Location (or reason for not reporting) |
| --- | --- | --- |
| **Title and abstract** |  |  |
| [1a. Indicate the study’s design](https:/resources.equator-network.org/reporting-guidelines/strobe/items/title-abstract-indicate-study-design.html?utm_source=strobe&utm_medium=checklist&utm_campaign=1_1) | Indicate the study’s design with a commonly used term in the title or the abstract. | Title, Page 1; Abstract, Methods, Page 1 |
| [1b. Abstract](https:/resources.equator-network.org/reporting-guidelines/strobe/items/abstract.html?utm_source=strobe&utm_medium=checklist&utm_campaign=1_1) | Provide in the abstract an informative and balanced summary of what was done and what was found. | Abstract, Results and Conclusion, Page 1 |
| **Introduction** |  |  |
| [2. Background / rationale](https:/resources.equator-network.org/reporting-guidelines/strobe/items/background-rationale.html?utm_source=strobe&utm_medium=checklist&utm_campaign=1_1) | Explain the scientific background and rationale for the investigation being reported. | Introduction, Pages 3-4 |
| [3. Objectives](https:/resources.equator-network.org/reporting-guidelines/strobe/items/objectives.html?utm_source=strobe&utm_medium=checklist&utm_campaign=1_1) | State specific objectives, including any prespecified hypotheses. | Introduction, Page 4 |
| **Methods** |  |  |
| [4. Study design](https:/resources.equator-network.org/reporting-guidelines/strobe/items/study-design.html?utm_source=strobe&utm_medium=checklist&utm_campaign=1_1) | Present key elements of study design early in the paper. | Methods, Study design and participants, Page 5 |
| [5. Setting](https:/resources.equator-network.org/reporting-guidelines/strobe/items/setting.html?utm_source=strobe&utm_medium=checklist&utm_campaign=1_1) | Describe the setting, locations, and relevant dates, including periods of recruitment, exposure, follow-up, and data collection. | Methods, Study design and participants, Page 5 |
| [6a. Eligibility criteria](https:/resources.equator-network.org/reporting-guidelines/strobe/items/eligibility-criteria.html?utm_source=strobe&utm_medium=checklist&utm_campaign=1_1) | **Cohort study:** Give the eligibility criteria, and the sources and methods of selection of participants. Describe methods of follow-up. **Case-control study:** Give the eligibility criteria, and the sources and methods of case ascertainment and control selection. Give the rationale for the choice of cases and controls. **Cross-sectional study:** Give the eligibility criteria, and the sources and methods of selection of participants. | Methods, Study design and participants, Page 5 |
| [6b. Matching criteria](https:/resources.equator-network.org/reporting-guidelines/strobe/items/matching-criteria.html?utm_source=strobe&utm_medium=checklist&utm_campaign=1_1) | **Cohort study:** For matched studies, give matching criteria and number of exposed and unexposed. **Case-control study:** For matched studies, give matching criteria and the number of controls per case. | Not applicable |
| [7. Variables](https:/resources.equator-network.org/reporting-guidelines/strobe/items/variables.html?utm_source=strobe&utm_medium=checklist&utm_campaign=1_1) | Clearly define all outcomes, exposures, predictors, potential confounders, and effect modifiers. Give diagnostic criteria, if applicable. | Not applicable |
| [8. Data sources / measurement](https:/resources.equator-network.org/reporting-guidelines/strobe/items/data-sources-measurement.html?utm_source=strobe&utm_medium=checklist&utm_campaign=1_1) | For each variable of interest give sources of data and details of methods of assessment (measurement). Describe comparability of assessment methods if there is more than one group. | Methods, Study design and participants, Page 5 |
| [9. Bias](https:/resources.equator-network.org/reporting-guidelines/strobe/items/bias.html?utm_source=strobe&utm_medium=checklist&utm_campaign=1_1) | Describe any efforts to address potential sources of bias. | Methods, Study design and participants, Page 5 |
| [10. Study size](https:/resources.equator-network.org/reporting-guidelines/strobe/items/study-size.html?utm_source=strobe&utm_medium=checklist&utm_campaign=1_1) | Explain how the study size was arrived at. | Methods, Study design and participants, Page 5 |
| [11. Quantitative variables](https:/resources.equator-network.org/reporting-guidelines/strobe/items/quantitative-variables.html?utm_source=strobe&utm_medium=checklist&utm_campaign=1_1) | Explain how quantitative variables were handled in the analyses. If applicable, describe which groupings were chosen, and why. | Methods, Survey, Page 5 |
| [12a. Statistical methods](https:/resources.equator-network.org/reporting-guidelines/strobe/items/statistical-methods-description.html?utm_source=strobe&utm_medium=checklist&utm_campaign=1_1) | Describe all statistical methods, including those used to control for confounding. | Methods, Descriptive analysis and Inferential analysis, Pages 5-6 |
| [12b. Statistical methods – subgroups and interactions](https:/resources.equator-network.org/reporting-guidelines/strobe/items/statistical-methods-subgroups-interactions.html?utm_source=strobe&utm_medium=checklist&utm_campaign=1_1) | Describe any methods used to examine subgroups and interactions. | Methods, Descriptive analysis and Inferential analysis, Pages 5-6 |
| [12c. Statistical methods – missing data](https:/resources.equator-network.org/reporting-guidelines/strobe/items/statistical-methods-missing-data.html?utm_source=strobe&utm_medium=checklist&utm_campaign=1_1) | Explain how missing data were addressed. | Not applicable |
| [12di. Statistical methods – loss to follow-up](https:/resources.equator-network.org/reporting-guidelines/strobe/items/statistical-methods-loss-to-follow-up.html?utm_source=strobe&utm_medium=checklist&utm_campaign=1_1) | **Cohort study:** If applicable, describe how loss to follow-up was addressed. | Not applicable |
| [12dii. Statistical methods – matching cases and controls](https:/resources.equator-network.org/reporting-guidelines/strobe/items/statistical-methods-matching-cases-controls.html?utm_source=strobe&utm_medium=checklist&utm_campaign=1_1) | **Case-control study:** If applicable, explain how matching of cases and controls was addressed. | Not applicable |
| [12diii. Statistical methods – sampling strategy](https:/resources.equator-network.org/reporting-guidelines/strobe/items/statistical-methods-analytical-methods-sampling-strategy.html?utm_source=strobe&utm_medium=checklist&utm_campaign=1_1) | **Cross-sectional study:** If applicable, describe analytical methods taking account of sampling strategy. | Methods, Descriptive analysis and Inferential analysis, Pages 5-6 |
| [12e. Statistical methods – sensitivity analyses](https:/resources.equator-network.org/reporting-guidelines/strobe/items/statistical-methods-sensitivity-analyses.html?utm_source=strobe&utm_medium=checklist&utm_campaign=1_1) | Describe any sensitivity analyses. | Methods, Sensitivity analysis, Page 6 |
| **Results** |  |  |
| [13a. Participant numbers](https:/resources.equator-network.org/reporting-guidelines/strobe/items/participants-numbers.html?utm_source=strobe&utm_medium=checklist&utm_campaign=1_1) | Report the numbers of individuals at each stage of the study—e.g., numbers potentially eligible, examined for eligibility, confirmed eligible, included in the study, completing follow-up, and analysed; Consider use of a flow diagram. | Results, Description of respondents and coding experience, Page 7 |
| [13b. Participants – non-participation](https:/resources.equator-network.org/reporting-guidelines/strobe/items/participants-non-participation.html?utm_source=strobe&utm_medium=checklist&utm_campaign=1_1) | Give reasons for non-participation at each stage. | Not applicable |
| [13c. Participants – flow diagram](https:/resources.equator-network.org/reporting-guidelines/strobe/items/participants-flow-diagram.html?utm_source=strobe&utm_medium=checklist&utm_campaign=1_1) | Consider use of a flow diagram. | Not applicable |
| [14a. Descriptive data – participant characteristics](https:/resources.equator-network.org/reporting-guidelines/strobe/items/descriptive-data-participant-characteristics.html?utm_source=strobe&utm_medium=checklist&utm_campaign=1_1) | Give characteristics of study participants (e.g., demographic, clinical, social) and information on exposures and potential confounders. Present the information in a table. | Results, Description of respondents and coding experience, Page 7 |
| [14b. Descriptive data – missing data](https:/resources.equator-network.org/reporting-guidelines/strobe/items/descriptive-data-missing-data.html?utm_source=strobe&utm_medium=checklist&utm_campaign=1_1) | Indicate the number of participants with missing data for each variable of interest. | Not applicable |
| [14c. Descriptive data – follow-up time](https:/resources.equator-network.org/reporting-guidelines/strobe/items/descriptive-data-follow-up-time.html?utm_source=strobe&utm_medium=checklist&utm_campaign=1_1) | **Cohort study:** Summarise follow-up time—e.g., average and total amount. | Not applicable |
| [15. Outcome data](https:/resources.equator-network.org/reporting-guidelines/strobe/items/outcome-data.html?utm_source=strobe&utm_medium=checklist&utm_campaign=1_1) | **Cohort study:** Report numbers of outcome events or summary measures over time. **Case-control study:** Report numbers in each exposure category, or summary measures of exposure. **Cross-sectional study:** Report numbers of outcome events or summary measures. | Results, Description of respondents and coding experience, Page 7 |
| [16a. Main results](https:/resources.equator-network.org/reporting-guidelines/strobe/items/main-results.html?utm_source=strobe&utm_medium=checklist&utm_campaign=1_1) | Give unadjusted estimates and, if applicable, confounder-adjusted estimates and their precision (e.g., 95% confidence intervals). Make clear which confounders were adjusted for and why they were included. | Results, Quality of health records, Clinical coding process and quality, Pages 8-12 |
| [16b. Main results – category boundaries](https:/resources.equator-network.org/reporting-guidelines/strobe/items/main-results-category-boundaries.html?utm_source=strobe&utm_medium=checklist&utm_campaign=1_1) | Report category boundaries when continuous variables were categorised. | Not applicable |
| [16c. Main results – risk](https:/resources.equator-network.org/reporting-guidelines/strobe/items/main-results-risk.html?utm_source=strobe&utm_medium=checklist&utm_campaign=1_1) | If relevant, consider translating estimates of relative risk into absolute risk for a meaningful time period. | Not applicable |
| [17. Other analyses](https:/resources.equator-network.org/reporting-guidelines/strobe/items/other-analyses.html?utm_source=strobe&utm_medium=checklist&utm_campaign=1_1) | Report other analyses done—e.g., analyses of subgroups and interactions, and sensitivity analyses. | Results, Inferential analysis, Pages 12 |
| **Discussion** |  |  |
| [18. Key results](https:/resources.equator-network.org/reporting-guidelines/strobe/items/key-results.html?utm_source=strobe&utm_medium=checklist&utm_campaign=1_1) | Summarise key results with reference to study objectives. | Discussion, Pages 13-14 |
| [19. Limitations](https:/resources.equator-network.org/reporting-guidelines/strobe/items/limitations.html?utm_source=strobe&utm_medium=checklist&utm_campaign=1_1) | Discuss limitations of the study, taking into account sources of potential bias or imprecision. Discuss both direction and magnitude of any potential bias. | Discussion, Strengths and limitations, Page 16 |
| [20. Interpretation](https:/resources.equator-network.org/reporting-guidelines/strobe/items/interpretation.html?utm_source=strobe&utm_medium=checklist&utm_campaign=1_1) | Give a cautious overall interpretation considering objectives, limitations, multiplicity of analyses, results from similar studies, and other relevant evidence. | Discussion, Implications, Page 17 |
| [21. Generalisability](https:/resources.equator-network.org/reporting-guidelines/strobe/items/generalisability.html?utm_source=strobe&utm_medium=checklist&utm_campaign=1_1) | Discuss the generalisability (external validity) of the study results. | Discussion, Implications, Page 17 |
| **Other information** |  |  |
| [22. Funding](https:/resources.equator-network.org/reporting-guidelines/strobe/items/funding.html?utm_source=strobe&utm_medium=checklist&utm_campaign=1_1) | Give the source of funding and the role of the funders for the present study and, if applicable, for the original study on which the present article is based. | Declarations, Funding, Page 19 |
